# Corneal Epithelial Tissue Engineering Strategy Based on Cell Viability Optimization: A Review and Prospects

**DOI:** 10.3390/bioengineering12111175

**Published:** 2025-10-29

**Authors:** Guoguo Tang, Miaomiao Chi, Yang Zhai, Rongmei Peng, Jing Hong

**Affiliations:** 1 Department of Ophthalmology, Peking University Third Hospital, Beijing 100191, China; 2010301336@bjmu.edu.cn (G.T.); miaoc001@126.com (M.C.); zyangdoct@163.com (Y.Z.); 2 Beijing Key Laboratory of Restoration of Damaged Ocular Nerve, Beijing 100191, China

**Keywords:** corneal epithelium, tissue engineering, limbal stem cell deficiency, biomaterials

## Abstract

Corneal transplantation is often considered the last resort for severe corneal epithelial disorders, especially limbal stem cell deficiency (LSCD). Tissue engineering offers novel strategies to mitigate the shortage of corneal transplant donors. However, low cell viability and compromised functionality in tissue engineering represent a major challenge. In this review, we describe the key characteristics required for corneal epithelium bioscaffolds. We summarize the research progress centered on optimizing cell activity and functionality in the past 10 years from four key perspectives: the sourcing of cells, seed cell pretreatments, biomaterial optimization, and engineered culture system innovation. The sources, isolation, and induction methods of seed cells are described, and the advantages and disadvantages of existing clinical treatment methods are compared. Furthermore, we compare existing clinical therapies and summarize promising seed cell pretreatment strategies for the first time. Several innovative engineered cell culture systems are exhibited as well. We demonstrated how to preserve cell viability through bioscaffold stiffness modulation, topographic design, and application of innovative fabrication techniques. Finally, we propose a personalized and precise regeneration strategy based on high-resolution images, digital modeling, bioprinting, and machine learning.

## 1. Introduction

The human cornea is a transparent, avascular, dome-shaped tissue covering the front part of the eye. It has dense sensory nerves and acts as a protective barrier [1]. Serving as the primary defense against environmental hazards, it protects intraocular structures from mechanical injury, radiation, microorganisms, and chemicals. As the outermost refractive element of the visual system, a healthy cornea accounts for about two-thirds of the eye’s total refractive power and plays a vital role in maintaining clear vision [2]. Blindness caused by corneal diseases is among the top five causes of blindness worldwide [3], accounting for over 5% of all cases [4]. In Asia, the prevalence of severe visual impairment and blindness due to corneal pathologies reaches 0.38% [5]. Corneal epithelial injuries arise from diverse etiologies, encompassing both enhanced destructive mechanisms, such as chemical burns, mechanical trauma, and infectious destruction [6], and compromised defensive mechanisms like exposure keratitis [7], neurotrophic keratitis [8], and severe dry eye syndrome [9], as well as systemic autoimmune disorders [10]. Under homeostatic conditions, limbal epithelial stem cells (LESCs) residing in the Palisades of Vogt maintain corneal transparency and structural continuity through self-renewal and epithelial repair mechanisms [11]. When congenital or acquired factors damage LESCs and their niche, epithelial self-renewal capacity is compromised. The barrier function mediated by the limbal architecture and its cellular components becomes impaired, leading to persistent pathological wound healing responses characterized by invasive inflammatory fibrovascular pannus formation, which was clinically defined as limbal stem cell deficiency (LSCD) [11]. Current clinical interventions for corneal epithelial injuries include topical and systemic pharmacotherapy [12], therapeutic contact lens wear [13,14], and amniotic membrane transplantation [15,16]. If basement membrane and stromal damage is involved, corneal transplantation may be indicated [17]. Once LSCD occurs, conventional keratoplasty often proves insufficient, necessitating transplantation of limbal tissue or ex vivo expanded LESCs to reconstitute the stem cell reservoir [18].

Corneal transplantation is often considered the last resort for severe corneal epithelial disorders. However, its clinical application is hindered by several limitations, including low donor survival rates, immune rejection, the necessity for long-term medication, and, most notably, donor shortage [19]. With the advancement of regenerative medicine, biotechnology-based repair strategies have achieved significant breakthroughs at the molecular, cellular, tissue, and organ levels. Regenerative medicine-based therapies for corneal epithelial repair encompass various technologies, such as tissue engineering, cell therapy, and gene therapy. Gene therapy, through gene-editing techniques and suitable delivery vectors, holds potential for curing corneal blindness by enabling non-immunogenic, safe, and sustained therapeutic responses. However, most current studies remain at the stage of animal experiments and early clinical trials. Its clinical translation still faces challenges related to safety, delivery efficiency, and long-term efficacy [20]. Cell therapy has shown promise in repairing corneal epithelial injuries; however, its effectiveness is constrained by low cell engraftment and survival rates, as well as limitations in cell sourcing and expansion [21]. Tissue engineering aims to reconstruct, repair, or replace damaged human tissues and organs through the integration of cells, scaffold materials, and bioactive factors, demonstrating significant potential for corneal epithelial regeneration [22]. It offers novel strategies to mitigate the shortage of corneal transplant donors.

**Figure 1:**
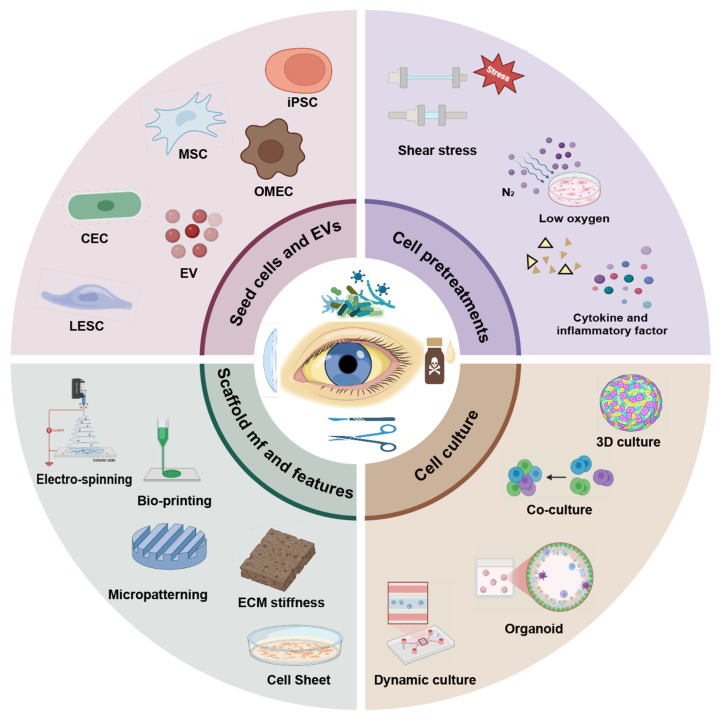
Tissue engineering strategies centered on optimizing cell activity and functionality. EVs, extracellular vesicles; CEC, corneal epithelial cell; MSC, mesenchymal stem cell; iPSC, induced pluripotent stem cell; OMEC, oral mucosal epithelial cell; LESC, limbal epithelial stem cell; mf, manufacture; ECM, extracellular matrix; 3D, three-dimensional.

Low cell viability and compromised functionality in tissue engineering represent a major challenge. The decline in cellular performance can be attributed to multiple factors, including the source and quality of seed cells, the properties of biological scaffolds, and limitations in culture systems. During in vitro expansion of LESCs, increased passaging leads to a significant reduction in the proportion of stemness markers such as p63 [23]. In addition, poor cell–cell and cell–extracellular matrix (ECM) interactions during transplantation can lead to the rapid loss of transplanted cells [24]. Synthetic scaffolds may further compromise cell survival, as certain materials are prone to eliciting inflammatory and fibrotic responses [25]. In bio-fabrication processes, such as three-dimensional (3D) bioprinting, exposure to environmental stressors, including ultraviolet light, reactive chemicals, and mechanical forces, often impairs cell viability and function [26]. Additionally, corneal epithelial cells (CECs) are highly sensitive to substrate stiffness; stiffer scaffolds activate mechano-transduction factors such as YAP and upregulate differentiation-related signaling pathways, including BMP4 [27]. Cellular vitality and function are coregulated by chemical, mechanical, and metabolic signals. Some tissue engineering approaches rely on static culture conditions, which fail to incorporate physiological cues native to the corneal epithelium, such as tear-induced shear stress. This often results in cells with lost polarity, low functional protein expression, and ineffective barrier formation [28]. In response to these challenges, recent studies have proposed various tissue engineering strategies centered on optimizing cell activity and functionality. This review summarizes advances from four key perspectives (Figure 1), namely, the sourcing of cells, seed cell pretreatments, biomaterial optimization, and engineered culture systems innovation, covering both currently applied and promising future technologies for corneal epithelial reconstruction.

## 2. Structure and Function of Corneal Epithelium

**Figure 2:**
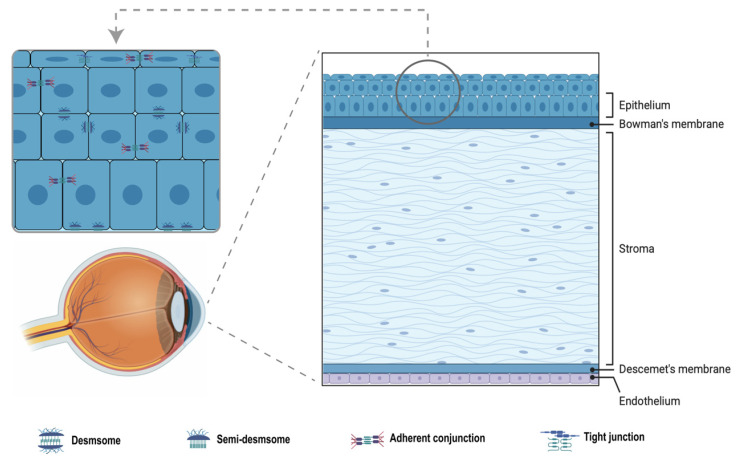
Cornea structure and cell junction between corneal epithelial cells.

The cornea consists of three main layers—the epithelium, stroma, and endothelium—separated by Bowman’s and Descemet’s membranes, respectively (Figure 2). The corneal epithelium is approximately 50 μm thick in humans, made up of 4–6 layers of non-keratinized stratified squamous epithelial cells [29,30,31]. Resting on the supportive stroma, the epithelium interacts with both the external environment and the underlying stromal tissue to maintain corneal transparency [32]. On average, the lifespan of epithelial cells is 7–10 days [33]. Human corneal epithelium undergoes continuous daily changes. Its self-renewal capacity relies on LESCs. LESCs represent a quiescent cell population with high proliferative potential [34], which is capable of both self-renewal and committed differentiation [35]. The Palisades of Vogt, a pigmented region at the corneal–conjunctival junction, forms anatomically distinct crypts that extend deeply into the stromal matrix, increasing the surface area to accommodate greater LESC numbers. LESCs are divided into two distinct subpopulations: active inner LESCs maintain corneal epithelial homeostasis, while quiescent outer populations contribute to the formation of the boundary between the cornea and conjunctiva [36].

CECs’ rapid turnover and intercellular junctions constitute the most important physical barrier against harmful external substances and pathogenic microorganisms. When these functions are normal, infectious inflammation rarely occurs. Histologically, the corneal epithelium can be divided into three layers from outer to inner, namely, the superficial layer, wing layer, and basal layer, each featuring specialized intercellular connections (Figure 2). In the superficial layer, squamous cells form a high-resistance semipermeable membrane through tight junctions, sealing paracellular spaces to create a selective barrier that regulates molecular and ionic diffusion. This structure ensures directional substance transport while preventing tear permeation into the stroma. These junctional complexes consist of cytoplasmic junctional proteins, such as the ZO family proteins ZO-1, ZO-2, and ZO-3, as well as transmembrane proteins occludin and claudin [37]. Desmosomes interconnect wing cells with each other and with superficial layer cells, providing critical mechanical support to the cornea. Basal cells anchor to the basement membrane via hemidesmosomes, securing the epithelium to the stroma [38]. Adherens junctions, mediated by E-cadherin and α-catenin [39], further connect all layers, playing essential roles in wound healing and tissue remodeling [40]. Barrier dysfunction occurs in diverse pathological contexts, including dry eye syndrome, infectious keratitis [41], diabetes [42], trauma, surgical injury, drug toxicity, and genetic disorders. Under in vitro tissue engineering conditions, the absence of native microenvironments and physiological signals leads to reduced expression of junctional proteins, loss of cell polarity, and weakened intercellular connections, resulting in widened gaps between cells [43]. In practical construction processes, the formation of tight junctions can be influenced by multiple factors, such as cell culture conditions and medium composition. Constructing a biomimetic ECM through nanoscale patterning and 3D culture systems to convey adhesion signals is critical for the proper formation of intercellular junctions. Corneal transparency is crucial for maintaining normal vision, relying on the cornea’s unique structure and molecular composition. The corneal stroma, which comprises approximately 90% of corneal thickness, consists of collagen fibers with uniform diameter and spacing, arranged in an extremely regular pattern. This structural foundation of corneal transparency minimizes light scattering, allowing visible light to pass through the cornea with minimal loss [44]. Corneal epithelial and endothelial cells are equally critical in regulating water balance to prevent stromal swelling [45]. In addition to the inherent transparency of scaffold materials required for minimizing light scattering, a critical challenge lies in promoting the formation of a stratified, compact, and uniform architecture by corneal epithelial cells on the scaffolds [46]. Furthermore, maintaining stem cell phenotypes after transplantation and suppressing neovascularization are essential for preserving visual quality [47]. Ideal tissue-engineered constructs for corneal epithelial repair should possess adequate mechanical strength, favorable biocompatibility, and high light transmittance. They should support cell adhesion and growth while preserving optical clarity through optimized microstructural organization, controlled hydration, physiologically relevant 3D cell distribution, and complete cell function [48].

## 3. Seed Cell Resources and Cell-Free Strategies

CECs and LESCs represent the optimal cell sources for seeding; however, their limited availability remains a constraint. In current research, stem cells have emerged as a promising alternative. Stem cells are endowed with excellent viability, enhanced proliferative capacity, and robust migratory potential, ensuring a sufficient number of highly viable cell populations for bioscaffold seeding and ocular surface application. Commonly utilized stem cell types include mesenchymal stem cells (MSCs) and induced pluripotent stem cells (iPSCs).

### 3.1. CECs and LESCs

Primary CECs represent the most direct cell source for transplantation. A Japanese research group developed an autologous transplantation strategy using cell sheets of cultured corneal epithelial cells grown on temperature-responsive culture dishes without carriers. Based on this technique, Nepic^®^ [49] was approved in Japan in 2020 for LSCD treatment. For patients with bilateral LSCD, alternative cell sources have been explored. In cases where no healthy limbal stem cells can be harvested from either eye, oral mucosal tissue serves as an important autologous cell source. The technique, known as cultured oral mucosal epithelial transplantation (COMET), involves harvesting a small biopsy of oral mucosa, expanding the epithelial cells in vitro, and transplanting the cultured sheet onto the ocular surface. In 2003, Nakamura et al. [50] first proposed the use of cultured human oral mucosal epithelial cells mounted on an amniotic membrane carrier for corneal epithelial reconstruction, and they published the clinical outcomes in 2004 [51,52]. In 2021 and 2022, oral mucosal epithelium-based corneal regenerative products, Ocural^®^ [53] and Sakracy^®^ [54], successfully completed clinical trials and received regulatory approval in Japan.

For patients with unilateral LSCD, autologous donor tissue containing LESCs can be harvested from the unaffected eye, offering higher success rates, fewer complications, and eliminating the need for lifelong systemic immunosuppression compared with allogeneic transplantation [55]. Conjunctival–limbal autograft (CLAU) harvests healthy conjunctival and limbal tissue, including the Palisades of Vogt, from the contralateral eye [56]. Due to its safety, long-term efficacy, and outstanding histocompatibility, CLAU remains the primary intervention for unilateral LSCD [57]. First implemented by Kenyon and Tseng in 1989 for unilateral LSCD management [58], CLAU was later modified by Chan et al. through integration with keratolimbal allograft (KLAL) to prevent donor conjunctiva from invading the recipient cornea [59], demonstrating superior graft survival compared to isolated KLAL [60]. To address donor tissue limitations, cultivated limbal epithelial transplantation (CLET) was developed [61], expanding autologous or allogeneic LESCs ex vivo on tissue-engineered substrates such as the amniotic membrane [62,63]. Compared with CLAU, CLET requires less donor limbal tissue, thereby reducing the risk of inducing LSCD in the donor eye [64]. Building on this, the first EU-approved stem cell-based drug, Holoclar^®^, was developed to treat moderate to severe LSCD caused by physical or chemical eye burns. It has not yet received FDA approval [65]. Alternative approaches include simple limbal epithelial transplantation (SLET) [66], which utilizes the amniotic membrane as an adhesive scaffold for transplanted LESCs. However, specialized culturing requirements and resource intensity restrict its clinical accessibility. More recently, a Harvard research team proposed cultivated autologous limbal epithelial cell (CALEC) therapy, drawing on extensive experience with ex vivo cultivated LESCs. The primary clinical indication for both CALEC and CLET is consistent, targeting the treatment of unilateral limbal stem cell deficiency (LSCD). Moreover, these two methods employ an analogous two-step strategy, which comprises in vitro expansion and subsequent in vivo transplantation. The CALEC manufacturing protocol was designed to optimize and standardize autologous LESC expansion for U.S. clinical trials, relying exclusively on FDA-approved materials, autologous cells, and highly reproducible methods for cell isolation and expansion while adhering to stringent quality control standards. The process emphasizes xeno-free, serum-free, and antibiotic-free conditions and employs a standardized two-step strategy: initial expansion of autologous LESCs in culture dishes until confluence, followed by transfer to a de-epithelialized amniotic membrane for further expansion [67]. The utilization of serum-free culture media eliminates batch-to-batch variability and mitigates risks such as immune rejection, infection, and potential toxic reactions. However, achieving precise control over specific components, including growth factors, cytokines, vitamins, and amino acids, imposes heightened technical requirements on researchers [68]. No serious adverse events were reported, supporting the efficacy and safety of CALEC transplantation in restoring corneal epithelial integrity [68].

### 3.2. MSC-Induced CECs

MSCs, initially identified in the bone marrow, represent a widely sourced class of adult multipotent stem cells derived from the bone marrow, amniotic membrane, umbilical cord, adipose tissue, and placenta. MSCs exhibit vigorous self-renewal ability and can differentiate into CECs through chemical induction or co-culture systems, efficiently expressing CEC markers, including CK3 and PAX6 [69]. These MSC-derived CECs demonstrate enhanced proliferative potential and functional intercellular junctions when cultured on tissue-engineered scaffolds [70]. Additionally, MSCs exhibit anti-angiogenic and anti-fibrotic properties that promote tissue regeneration [71]. Yao et al. [72] observed accelerated epithelial recovery and VEGF downregulation in alkaline burn rat models following subconjunctival BM-MSC injection. Fu et al. [73] discovered that human UCMSC implantation into the subconjunctival space promoted epithelial repair, reduced inflammation and neovascularization, and attenuated stromal disorganization of collagen and fibronectin. MSC-mediated immunomodulation further suppresses post-transplant inflammation, enhancing graft survival [74]. However, MSC therapy has limitations. Currently, MSCs are often derived from allogeneic sources, raising concerns of immune rejection and necessitating long-term immunosuppressive medication, which restricts their broader clinical application.

Notably, in addition to conventional BM-MSCs and HUMSCs, alternative MSC sources show therapeutic potential. For example, human oral mucosa stem cells (hOMSCs), derived from neural crest origin, can express embryonic stem cell (ESC) markers (TRA-2-49, SSEA-4, NANOG), MSC surface markers (CD73, CD90, CD105), and nestin [75]. They are easy to isolate and have age-independent viability, providing new options for bilateral LSCD. Sonia et al. [76] reported that hOMSCs can adhere to amniotic membranes with the expression of epithelial differentiation markers (CK3, CK19, P-cadherin, E-cadherin).

### 3.3. iPSC-Induced CECs

iPSCs demonstrate therapeutic potential for corneal repair. Chien et al. [77] loaded human corneal keratocyte-derived iPSCs into a thermo-gelling injectable amphipathic carboxymethyl-hexanoyl chitosan (CHC) nanoscale hydrogel. In an alkaline burn model, the iPSC/CHC hydrogel restored epithelial thickness through oxidative stress downregulation and endogenous CEC recruitment. iPSCs can be derived from patients’ somatic cells, thereby avoiding immune rejection risks and ethical concerns while stably expressing corneal epithelial markers. Yu et al. [78] co-cultured murine iPSCs with limbal stroma in the presence of bFGF, EGF, and NGF, observing successful differentiation into CEC-like cells with upregulated K12 and downregulated Nanog expression. Lee et al. [79] investigated the therapeutic effects of exosomes derived from iPSC-derived retinal organoids on corneal epithelial wound healing. Exosome-treated eyes showed significantly enhanced wound closures compared with controls at 24 h post-injury, while a substantial increase in cell proliferation and a decrease in inflammatory marker contents were observed. Exo-RO treatment targeted various pathways related to inflammation and cell proliferation, including the PI3K-Akt, TNF, MAPK, and IL-17 signaling pathways.

In 2016, Hayashi et al. [80] successfully generated self-forming two-dimensional organoids from iPSCs, termed self-formed ectoderm autonomous multizone (SEAM), which mimic whole-eye development. By isolating and expanding corneal epithelial-like cells from the ocular surface ectodermal zone of SEAMs, the team produced functional iPSC-derived corneal epithelial cell sheets. This work marked a milestone in iPSC-induced CEC application. Recently, researchers from Osaka University conducted the first human trial of iPSC-derived CECs. They transplanted iPSC-derived corneal epithelial cell sheets into four eyes with LSCD. During the 24-month follow-up, no serious adverse events, such as tumors or clinical rejection reactions, were observed. Ultimately, three patients achieved Stage IA remission and one patient achieved Stage IIB LSCD remission, with all eyes exhibiting improved visual acuity, reduced corneal opacity and epithelial defects, and alleviated subjective symptoms. The study preliminarily demonstrated the efficacy and safety of iPSC therapy for LSCD [81].

### 3.4. Extracellular Vesicles and Enucleate Cells

Extracellular vesicles (EVs) are nanoscale membranous particles that mediate intercellular communication. They are secreted by all cell types and carry diverse molecules, such as proteins, lipids, or nucleic acids. As EVs possess low immunogenicity and lack tumorigenic risk, they hold potential in cell-free strategies for corneal epithelial tissue engineering [82]. Unlike stem cells with stringent storage and viability requirements, EVs offer advantages in procurement, preservation, and transportation. MSC-derived EVs effectively promote the migration and proliferation of CECs, accelerate wound healing, and reduce inflammatory responses and scar formation [83]. Engineered EVs further optimize therapeutic outcomes. EVs loaded with specific miRNAs, such as miR-24-3p, miR-29b-3p, and miR-432-5p, improve corneal repair by modulating autophagy, inhibiting inflammatory pathways, and reducing collagen deposition [84,85]. Surface conjugation with targeting ligands enables precise cellular targeting. Other studies have combined exosomes with novel delivery systems, such as thermosensitive hydrogels, to sustain drug release and prolong their action time on the ocular surface [84]. Xu et al. [86] developed EV-functionalized gelatin methacryloyl (GelMA) hydrogels with 3D-distributed vesicles, enabling high-viability EVs to remain on the ocular surface for an extended period. By targeting PDCD4, this system promotes the proliferation of corneal epithelial and stromal cells while reducing the release of inflammatory factors. Although EVs circumvent cell culture challenges, their clinical translation faces persistent obstacles, including short ocular residence time and low bioavailability.

Enucleated cells represent a conceptually similar approach, wherein cells are deprived of their nuclei, thus losing the ability to proliferate or permanently engraft in the host while retaining functional organelles capable of energy production and protein synthesis. These anucleated cells serve as efficient and safe carriers for targeted therapeutic delivery. Wang et al. [87] employed density gradient centrifugation to generate enucleated cells from genetically engineered MSCs, which were designed to express chemokine receptors and endothelial-binding molecules. These modified, enucleated MSCs demonstrated the ability to actively home to diseased tissues and deliver therapeutic payloads, such as the anti-inflammatory cytokine IL-10, with high specificity. In mouse models of acute inflammation and pancreatitis, these enucleated carriers significantly enhanced drug delivery efficiency and reduced inflammation. They outperformed both unmodified MSCs and MSC-derived extracellular vesicles. Crucially, they eliminated risks associated with cell proliferation or long-term engraftment. This strategy offers a promising and safe platform for targeted therapy in inflammatory diseases.

The following table (Table 1) summarizes several major technical strategies of corneal epithelial regeneration.

## 4. Cell Pretreatment

Cellular viability and functionality are critically influenced by culture conditions. For specific cell types, pretreatment methods, such as cytokine stimulation, hypoxia induction, 3D culture, and heat shock, have been shown to enhance cells’ tolerance to stress during transplantation and engineering processes.

### 4.1. Physical Pretreatment

Reconstructing the complex in vivo physical microenvironment in vitro is the most straightforward and well-established approach. The cornea is constantly subjected to shear forces generated by blinking and tensile forces caused by intraocular pressure. Hampel et al. [88] cultured CECs in vitro with adjustable flow to simulate the discontinuous shear stress applied by blinking. Significant changes in cell morphology and expression of cell adhesion proteins were found, which better replicated physiological conditions compared to conventional static culture. Molladavoodi et al. [89] identified shear preconditioning as pivotal for wound healing through cytoskeletal reorganization and enhanced migration/proliferation rates, and this phenomenon was more obvious under low shear stress (4 dyn/cm^2^). However, excessive shear proved detrimental. Utsunomiya et al. [90] observed elevated TGF-β1 expression and SMAD2 phosphorylation of CECs under high shear, correlating with reduced proliferation, delayed healing, and MMP9 activation, indicating the involvement of inflammatory pathways in excessive shear stimulation. On the other hand, unlike terminally differentiated CECs, stem cells often benefit from moderate hypoxic conditions [91]. Ma et al. [92] reported augmented proliferative capacity in mouse LESCs under in vitro hypoxic conditions (20 mL/L O_2_ and 50 mL/L CO_2_). For MSCs, normoxic culture induces oxidative DNA damage and senescence [93], compromising viability [94]. Conversely, 2–5% O_2_ promotes MSC proliferation, survival, migration, and stemness maintenance [95]. Hypoxic preconditioning greatly enhances cell resistance to subsequent damage, protecting cells from oxidative damage and cellular aging by stabilizing HIF-1α [96]. Additionally, 3D culture systems simulate corneal spatial microenvironments significantly, enhancing seeded cell vitality, which will be discussed in detail in subsequent sections.

### 4.2. Biochemical Pretreatment

Preconditioning seed cells with inflammatory or cytokine stimuli remains relatively underexplored in corneal epithelial tissue engineering. Wang et al. [97] pre-treated rabbit LESCs with TNF-α and collagenase IV, generating 3D LSC spheroids with enhanced proliferative capacity while maintaining stemness in embryonic stem cell-conditioned medium. Preconditioned LSCs exhibited elevated spheroid formation efficiency, reduced apoptosis, significantly upregulated Oct4 and β-catenin expression, and improved scaffold adhesion. Additionally, inflammatory cytokines, such as TNF-α, INFγ, and IL-1β, have been extensively studied for pre-activating stem cells [98] with improved therapeutic effects confirmed in models of liver cirrhosis [99], periodontitis [100], and skin injury [101]. Moderate cytokine preconditioning may thus augment corneal epithelial seed cell proliferation and anti-inflammatory ability, potentially promoting better corneal regeneration.

## 5. Engineering-Oriented Culture Systems

Expanding a large number of viable cells through engineered cultivation systems before transplantation represents a critical step in tissue regeneration. Current culture strategies are rapidly evolving from traditional two-dimensional static systems toward the construction of biomimetic microenvironments. Conventional culture models fail to recapitulate the physicochemical properties and complex cell–cell interactions in vivo. In contrast, innovative culture strategies that integrate dynamic mechanical stimulation, spatially controlled co-culture, and 3D microenvironmental reconstruction are driving substantial advances in the maintenance of seed cell viability, functional differentiation, and biomimetic tissue organization. Indeed, 3D culture enables more natural cell–cell and cell–matrix interactions than static systems, facilitating the formation of stratified structures and supporting barrier function [102]. However, a major limitation is that diffusion of nutrients and oxygen becomes restricted within larger 3D cellular aggregates or scaffolds, often leading to central necrosis or functional impairment over extended culture periods [103]. This has spurred the development of dynamic perfusion culture systems [104], which simulate physiological shear forces on the ocular surface and improve nutrient supply and waste removal [105]. Co-culture systems further introduce a biological dimension of cellular communication. By incorporating multicellular communities, they accurately replicate the niche signaling and crosstalk present in native tissues. The integration of 3D structural support, dynamic perfusion stimuli, and multicellular interactions has given rise to a “structural, mechanical, and biological” biomimetic microenvironment, which is emerging as a new paradigm in engineered tissue cultivation.

### 5.1. Dynamic Culture

Dynamic cell culture facilitates cell growth under physiologically relevant conditions by exerting continuous mechanical stimuli, such as fluid shear and tension, as well as enabling the continuous exchange of nutrients and signaling molecules. This includes various methods such as microfluidic platforms and dynamic air–liquid interface (ALI) culture.

Microfluidics represents a pivotal dynamic culture technology. It employs micrometer-scale channels (10–1000 μm) to manipulate fluids with precise control over flow velocity, pressure, and compositional gradients. Abdalkader et al. [106] analyzed the shear stress during blinking and then cultured CECs on a central porous membrane. At day 7, the cells formed a barrier with high ZO-1 expression. Subsequent application of bidirectional flow in the upper channel and continuous flow in the lower channel for 24 h significantly enhanced cytokeratin 19 intermediate filament expression, indicating strengthened barrier function. In addition to cell culture, microfluidic cornea-on-a-chip is an important application, which enables physiologically relevant reconstruction of human corneal environments for preclinical drug evaluation in a time-saving and cost-effective manner. Bennet et al. [107] designed an integrated microfluidic device featuring dual PDMS channels separated by a porous polycarbonate membrane (5 μm pores), reconstructing the corneal epithelium, basement membrane, and Bowman’s layer, enabling further investigation of the dynamic transport of drugs through the corneal epithelial barrier. In addition, Lee et al. [108] established a high-throughput microfluidic wound-healing model using CECs, demonstrating artemisinic acid’s therapeutic effects with substantially reduced reagent consumption and processing time compared to conventional assays.

ALI culture is another method for simulating blinking. Wu et al. [109] utilized a decellularized porcine corneal stroma scaffold, combining static culture, perfusion culture, and dynamic ALI culture, to construct an engineered pre-lamellar corneal tissue for transplantation. After 5 days of static culture, the corneal grafts were placed in a dynamic perfusion high-oxygen-pressure culture medium with oxygen exchange devices on both sides for 2 days, followed by a final culture for 3 days under conditions of air–liquid exchange at the top and perfusion culture at the bottom to obtain the final material for transplantation. Compared to static controls, the dynamic ALI constructs demonstrated superior epithelial differentiation, achieving native-level transepithelial electrical resistance, light transmittance, and surface elastic modulus. The post-lamellar keratoplasty construct in rabbits exhibited optical properties and wound healing kinetics comparable to native tissue.

There are other emerging technologies that show significant potential for corneal epithelial tissue engineering. Surface acoustic wave (SAW) devices generate microscale circulation in conventional culture dishes, enhancing nutrient and waste exchange and boosting U-937 cell proliferation by 36% without altering cell morphology [110]. SAW enables rapid, uniform cell and particle infiltration, reducing seeding time while improving spatial distribution [111]. Integrated monitoring systems, like microfluidic-embedded biosensors and corneal bioreactors, can be applied for real-time viability tracking, combined with intelligent control and automated feeding strategies to dynamically adjust culture conditions.

### 5.2. Co-Culture

Co-culturing corneal epithelial cells with associated cell types, such as stem cells and stromal cells, recapitulates native cell–cell and cell–matrix interactions. These interactions provide structural support, signal exchange, and material transfer. Through the synergistic effect between cells, cell survival and proliferation are improved, and cell function is maintained.

Co-culturing CECs with matrix cells in 3D is a common approach. Gosselin et al. [112] established a 3D co-culture system on silk fibroin membranes under ALI conditions with topographical optimization. Combining corneal stromal stem cells and CECs yielded stratified epithelial and stromal structures. Compared to simply corneal stromal cells or CECs, the differentiation and growth of both types were more complete, and cell proliferation was more vigorous. Co-culture not only allows direct cell–cell contact but also enhances intercellular communication. Matrix cells are capable of secreting various soluble factors, which significantly stimulate DNA synthesis and proliferation in epithelial cells [113]. They can also regulate ECM component synthesis, thereby bettering the cellular microenvironment [114]. Co-culture of CECs with stem cells has a bidirectional effect. Soleimanifar et al. [115] co-cultured conjunctiva-derived MSCs with CECs in supplemented hormonal epithelial medium. The MSCs enhanced epithelial proliferation while the epithelial cells provided a differentiation-favorable microenvironment for MSC phenotypic commitment.

LESCs are anchored at the basement membrane, with their maintenance critically dependent on the stem cell niche. Supportive cells, including limbal stromal cells and melanocytes, play decisive roles in LESC self-renewal, proliferation, and differentiation [116]. In vitro reconstitution of the limbal stem cell niche significantly enhances LESC viability and stemness retention. Ahmadiankia et al. [117] observed reduced apoptosis and significantly increased viability when co-culturing LESCs with limbal fibroblasts and mouse embryonic fibroblasts. Nam et al. [118] demonstrated that MSCs enhanced LESC growth through secreted growth factors and transforming growth factor β-induced protein (TGFBIp) within the ECM. The proportion of p63+/ABCG2+ LESCs was increased. Melanocytes promote stratified LESCs growth while maintaining an undifferentiated state in both 2D and 3D environments [119]. Co-culture systems also drive multipotent stem cells and amniotic epithelial cells toward corneal epithelial differentiation, evidenced by the expression of epithelial markers CK3, CK12, and PAX6 [120]. However, the current co-culture cell composition remains relatively simple. It may be worthwhile to incorporate in vivo cellular ratios of the limbal niche through gradient seeding strategies, spatially pre-positioning cells in distinct scaffold regions to emulate niche heterogeneity.

### 5.3. Three-Dimensional Culture

Conventional 2D culture systems often fail to replicate physicochemical properties; thus, cells exhibit significant deviations between their in vitro and in vivo counterparts [121]. Cells tend to grow in a flattened manner, leading to altered polarity, differentiation, and metabolism. Such deviations may disrupt critical cellular interactions and decrease stemness. Three-dimensional culture can provide different mechanical stimuli. This method holds advantages in simulating tissue–tissue interfaces and mechanical microenvironments, bridging the gap between 2D cell culture and animal models [122]. Carter et al. [123] characterized MSC secretome profiles under 2D versus 3D conditions, revealing that 3D-cultured MSCs promote CECs proliferation through differential secretome signatures. Corneas treated with 3D-cultured MSCs exhibited accelerated re-epithelialization and reduced scar formation.

Cells are typically subjected to 3D culture through four methodologies: suspension on non-adherent surfaces or in bioreactors, embedding in hydrogels, placement on mechanical scaffolds, such as tissue culture inserts for air–liquid interface culture, and seeding onto scaffolds and microcarriers [124]. The application of 3D-printed bioscaffolds is a current trend. Wu et al. [125] fabricated collagen/gelatin/alginate hydrogel structures loaded with CECs using extrusion-based printing. The hydrogel network exhibited large pore sizes and stability. By adjusting the sodium citrate/sodium alginate molar ratio, the degradation time of scaffolds can be controlled, with cell survival rates exceeding 90% and increased CK3 expression.

Static 3D cultures suffer from limited nutrient diffusion and metabolite clearance in central regions, often causing necrosis or maturation heterogeneity within organoids. Consequently, integrating dynamic cultivation into 3D systems arises. Li et al. [126] applied perfusion culture bioreactors and 3D spheroid culture. These CECs showed elevated proliferative capacity and tightened cell–cell junctions with abundant surface microvilli. This approach facilitated CEC monolayer formation on decellularized corneal scaffolds and collagen sheets, accompanied by de novo ECM synthesis.

In practical applications, integrated cultivation strategies are increasingly employed, from which organoids are derived. Organoids represent self-assembling miniaturized tissues that recapitulate native organ structures and functions without exogenous scaffolds. The integration of “3D + co-culture + dynamic culture” has become a mainstream trend, offering options for exploring in vitro physiological processes and establishing disease models; it can also serve as a tissue source for corneal epithelial replacement. Current limbal organoids maintain viability for >1 month ex vivo and effectively serve as direct cell sources for corneal epithelial transplantation in rabbit LSCD models [127].

## 6. Biomimetic Scaffold and Manufacture Processes

The corneal epithelial basement membrane (BM), Bowman’s layer, and stroma play essential roles in maintaining normal structure and function. They not only provide structural support but also regulate epithelial growth, differentiation, and migration through their mechanical and biochemical properties [128]. The BM is primarily composed of ECM proteins, such as type IV collagen, laminin, nidogen, and heparan sulfate proteoglycans. These proteins form a thin, dense meshwork that offers stable attachment sites and biological signals for epithelial cells [129]. The BM exhibits certain mechanical strength and viscoelasticity, with an average elastic modulus of 7.5 ± 4.2 kPa [130], while that of Bowman’s layer is 109.8 ± 13.2 kPa. Together with the orthogonally arranged stromal layer, they provide excellent mechanical strength and light transmission, withstand mechanical stresses generated by epithelial activities, and mediate mechano-transduction signaling. Under electron microscopy, the BM displays a nanoscale undulating surface topography and a heterogeneous pore network [131]. Its unique topographic and topological characteristics directly influence cell migratory behavior. It is essential to not only incorporate key BM components but also holistically consider the mechanical properties, topological features, and biochemical signaling attributes of both the BM and stroma. This section reviews advancements in stiffness modulation, topographic design, and emerging fabrication techniques.

### 6.1. Stiffness-Regulating Mechanical Properties

Cells actively perceive extracellular matrix stiffness cues, which critically regulate growth, proliferation, migration, immunity, and apoptosis. Analogously, scaffold stiffness in tissue engineering profoundly influences CEC viability. Gouveia et al. [27] demonstrated that LESCs cultured on soft collagen scaffolds (~15 kPa) exhibited elevated proliferation and stratification. They expressed higher levels of proliferative markers (Ki67, β-catenin) and LESC markers (ΔNp63, ABCG2, CK15). Conversely, stiffer substrates (~65 kPa) downregulated stemness markers, promoted differentiation, and activated mechano-transduction factor YAP. Stiffness variations further modulated cytoskeletal proteins, like phospho-cofilin, profilin, focal adhesion kinase, and vinculin, impacting cell morphology, adhesion, migration, and endocytosis. Sun et al. [132] utilized methanol-treated silk fibroin membranes with tunable substrate stiffness to culture CECs. It was observed that LESCs cultured on silk membranes of varying stiffnesses (ranging from 13.4 ± 5.4 kPa to 445.4 ± 138.7 kPa) exhibited distinct mechanosensitive responses, including differences in cell spreading area, nuclear localization of YAP, and increased actin cytoskeletal tension, suggesting a regulatory role of substrate mechanics in ocular surface repair.

A review of existing studies indicates that many engineered scaffolds exhibit stiffness values reaching tens or even hundreds of kPa, which significantly exceeds the physiological range of CECs and is therefore suboptimal for their growth and function. In the design of bioscaffolds, it is essential to holistically consider factors such as the mechanical properties of the material and cellular viability. Maintaining substrate stiffness within a physiologically appropriate range remains a critical area requiring continued optimization by researchers.

### 6.2. Material Topography and Micropatterned Surfaces

Advancing bioprinting precision alongside photolithography and micro-imprinting technologies now enables the fabrication of highly biomimetic scaffolds [133]. It is now possible to form specific textured morphologies at the micron and nanometer levels. These topographic cues apply a sustained and uniform influence on cells. They provide a spatial anchor, transmitting mechanical signals through cell–matrix interactions to modulate cytoskeletal organization and subsequent gene expression [134]. The utilization of material topography can effectively enhance cell adhesion, proliferation, and maintain cellular pluripotency. Aboal-Castro et al. [135] employed femtosecond laser etching on 3D-printed poly (ε-caprolactone) scaffolds to create microgrooves (10/80 μm width) and micropits (25 μm diameter). Cells arranged in elongated forms along the grooves, with significantly enhanced cell adhesion, while cells on micro-pores and unpatterned surfaces helped maintain a polygonal shape. Lawrence et al. [136,137] cultured LESCs on a microgrooved silk fibroin membrane. An average increase in the adhesion rate of 36–54% was observed, equivalent to a more than 2-fold increase in the number of focal adhesions. The groove patterns not only clearly guided cell migration but also resulted in better cell stemness. Öztürk- Öncel et al. [138] functionalized polydimethylsiloxane (PDMS) with collagen IV and hyaluronic acid and then imposed white rose petal micropatterns. The patterned scaffold supported denser corneal endothelial monolayers with upregulated phenotypic markers, surface roughness, feature dimensions, and arrangement count. The height and spacing of microcolumn structures determine cell distribution and adhesion capacity. Microstructures with high surface free energy enhance cell number and viability [139]. Zhang et al. [140] established a highly biomimetic corneal model by integrating topographical and mechanical cues. Patterned silk films induced organized corneal cell architectures while imparting 3% dome-shaped strain, collectively elevating corneal cell and extracellular matrix marker expression.

### 6.3. Innovative Scaffold Manufacture Processes

#### 6.3.1. Electrospinning

**Figure 3:**
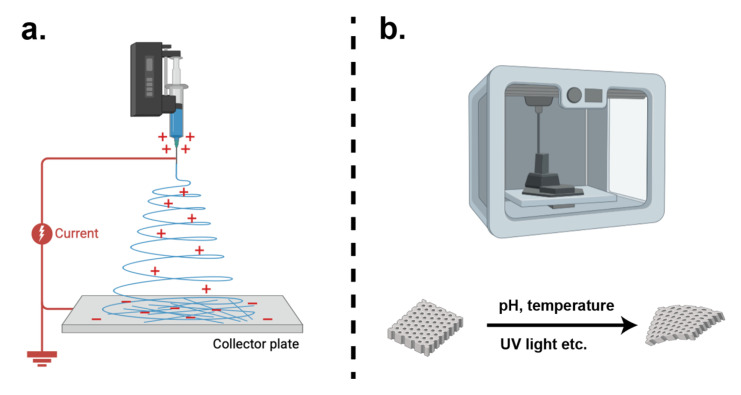
Pattern diagram for electrospinning and 4D bioprinting: (**a**) electrospinning; (**b**) 4D bioprinting.

Electrospinning utilizes high-voltage electrostatic fields to process polymer solutions or melts, depositing nanofibers with diameters less than 1000 nm onto a target substrate to form a spiderweb-like structure (Figure 3a). Commonly used substrates include gelatin, collagen [141], PLGA, etc. These scaffolds exhibit high porosity, substantial surface area, and tunable fiber dimensions. Such structural properties facilitate nutrient diffusion while supporting cellular adhesion, movement, proliferation, and differentiation. Electrospun nanofibers exhibit excellent mechanical properties as well [142]. Recent advancements integrate electrospinning with 3D printing, multimaterial composites, and surface modification technologies to further augment biomimetic properties.

In terms of material structure, conventional electrospun scaffolds are primarily suited for thin-layer planar structures, which are often inadequate for 3D spherical complexes like the eyeball, as cell distribution inside is inhomogeneous. Kim et al. [143] fabricated a radially patterned 3D hemispherical PCL/collagen nanofibrous mat via a modified rotating collector. The pore size ranged from 0.5 to 0.8 μm, facilitating substance diffusion and cell interactions. This 3D radial nanofiber scaffold demonstrated excellent capabilities in inducing cell morphogenesis and cell migration. Sandwich-structured electrospun constructs address multifaceted requirements, including cell growth, drug-controlled release, and transparency, by layering distinct materials. Arabpour et al. [144] designed a three-layer electrospun nanofiber scaffold composed of PLGA/collagen/PLGA. Aligned type I collagen nanofibers in the middle layer enhanced mechanical strength, while the outer PLGA layer improved biocompatibility and promoted cell adhesion. It exhibited higher strength and cell viability compared to traditional single-layer scaffolds. Similarly, Kong et al. [145] developed a hybrid structure combining PC collagen and electrospun PLGA pads, with excellent biocompatibility and mechanical properties.

Surface modification of electronspun scaffolds represents an innovative approach. Mahmood et al. [146] optimized electrospinning parameters to construct uniformly coated nanofiber scaffolds with Noggin, which demonstrated higher proliferation and wound healing rates when loaded with corneal epithelial cells. Jafar et al. [147] subjected PLGA nanofibers to plasma treatment, which significantly enhanced surface hydrophilicity and modulated surface charge without altering physical properties. The modified surfaces were further functionalized with plasma or fibronectin to promote 3D cell attachment, resulting in a substrate more conducive to the adhesion, proliferation, and growth of LESCs.

#### 6.3.2. Bioprinting

Bioprinting primarily relies on four types of techniques: inkjet, extrusion, laser-assisted, and lithography. Hydrogels, due to their biomimetic properties, are currently considered the most suitable biomaterial for bioink, enabling encapsulated live cells to adhere, proliferate, and differentiate. Compared to electrospinning, bioprinting places greater emphasis on macrostructural support [148]. Its high precision and robust architectural potential make it an indispensable method for manufacturing biological scaffolds. Nevertheless, bioprinted constructs demonstrate comparatively limited microstructural biomimicry. During the fabrication process, cells are subjected to various mechanical stresses and chemical stimuli, which impact cell viability and function through cell signaling and protein expression [149]. Preserving cell viability is a major challenge in bioprinting, especially in 3D printing.

Among various manufacturing types, extrusion is the most cell-friendly. Lower extrusion pressures and larger nozzle diameters typically minimize mechanical damage, though higher shape fidelity necessitates smaller nozzles. Regarding nozzle shape, a tapered one reduces pressure requirements at equivalent flow rates [150]. Wang et al. [151] observed diminished viability under elevated pressure/shear stress with small nozzles, while structured bioinks significantly mitigated these forces to improve survival rates. In inkjet bioprinting, pulse parameters and frequency modulate shear stress effects on viability. Wang et al. [152] achieved ~95% viability through optimized waveform design. Printing temperature and speed are also critical factors to consider. Ouyang et al. [153] systematically demonstrated that higher printing temperatures and lower gelatin concentrations enhance embryonic stem cell viability during 3D bioprinting, with viability inversely correlating exponentially with shear stress. Boularaouiet al. [154] further reported 7.8% and 6.6% viability increases in shear-preconditioned C2C12 myoblasts extruded through nozzles and needles, respectively, compared to nonconditioned cells. The following table (Table 2) summarizes the bioinks and printing parameters, as well as the corresponding cell viabilities, reported in the recent research that applies extrusion 3D printing to manufacture corneal equivalents.

Four-dimensional printing (Figure 3b), an advancement built upon three-dimensional printing technology, incorporates a temporal dimension, enabling printed constructs to undergo dynamic morphological or functional changes in response to external stimuli, such as pH, temperature, or light [164]. This approach addresses several limitations of conventional 3D printing, particularly in enhancing cell viability. Since 4D-printed scaffolds are designed to change predictably after printing under specific conditions, they can be fabricated using low temperatures and non-toxic crosslinking agents, especially in hydrogels and shape-memory polymers, thereby reducing cellular damage during manufacturing [165]. Furthermore, 4D-printed scaffolds can better adapt to the complex geometry and biomechanical environment of host tissues after implantation, mitigating mechanical mismatch and reducing the risk of graft failure [166]. An ideal bioscaffold should possess a well-designed porous structure to facilitate cell attachment, proliferation, differentiation, efficient nutrient and oxygen transport, and metabolic waste removal. Four-dimensional printing allows for the dynamic and post-fabrication modulation of pore architectures. For instance, Wang et al. [167] developed a temperature-responsive chitosan-based 4D-bioprinted stem cell carrier for repairing alkaline-burned corneas, which exhibited uniform pore size and significantly improved cell loading efficiency and regenerative outcomes. Moreover, by responding to physiological cues, such as pH, osmotic pressure, and mechanical stress, 4D-printed scaffolds can mimic dynamic curvature adjustments and microenvironmental changes in the cornea under varying physiological conditions. Song et al. [168] demonstrated that 4D-printed materials could adapt to ongoing changes in bone structure during tooth development and soft tissue remodeling, highlighting the broader potential of adaptive scaffolds in biomimetic tissue engineering.

Bioinks with higher proportions of natural components typically exhibit enhanced biocompatibility but inferior mechanical strength. Stepanovska et al. [169] improved collagen gel performance via sodium hydroxide surface modification, maintaining high cell viability while increasing elastic modulus. Moderate viscosity and shear-thinning behavior aid in cell protection and structural formation during printing. Excessive viscosity elevates extrusion pressure and cellular damage, whereas insufficient viscosity compromises shape fidelity. Incorporating nanocellulose or nano-crystal cellulose enhances shear-thinning properties and mechanical robustness without compromising viability, enabling complex architectural printing [170].

Machine learning offers efficient parameter optimization beyond large-scale empirical approaches. Limon et al. [171] integrated decision trees and clustering algorithms to predict the viscosity of bioinks with various components. Zhang et al. [172] developed a quantitative framework that integrated numerical simulation and support vector regression to assess alginate ink viscosity–shear stress profiles, predicting extrusion-induced cell viability based on wall shear stress magnitude and exposure duration.

There is often a lack of nutrients in the central part of the structure in 3D printing due to the limited diffusion distance of nutrients and oxygen. Scaffold geometry, material properties, and culture conditions collectively govern cellular and nutrient distribution. Shao et al. [173] engineered a dual-nozzle system to co-print cell-laden gelatin methacryloyl (GelMA) bioinks alongside sacrificial gelatin networks. Postprinting, reversible thermal cross-linking of GelMA allowed selective dissolution of gelatin to create perfusable nutrient channels. This architecture significantly enhanced cell viability compared to solid constructs, which progress over an extended culture. Other studies have incorporated components such as gelatin–CaO_2_ microspheres [174] and single-cell microalgae [175] through sustained oxygen release or photosynthesis.

### 6.4. Cell Sheets

Tissue engineering can be simply summarized as cells and biological scaffolds. If the application of extracellular vesicles represents a cell-free design in tissue engineering, then cell sheet technology represents a scaffold-free approach. Cell sheets eliminate exogenous material addition, whether natural or synthetic, thereby minimizing heterogeneity and providing seed cells with a native-like ECM. However, it also suffers from poor mechanical strength. Although temperature-responsive culture dishes facilitate intact sheet detachment, storage and handling challenges persist. Guo et al. [176] continuously cultured MSCs for 21 days using an expansion medium and an induced ECM medium, resulting in a cell sheet structure with elastin increased by 10,000-fold, a higher elastic modulus (1500 kPa), and a thicker structure (20.59 μm). When CS was transplanted into alkaline-burned corneas, significant therapeutic effects were observed.

## 7. Future Directions

Despite significant advances in optimizing cellular viability and functionality in corneal epithelial tissue engineering, several critical limitations remain. Patients with severe epithelial damage are faced with a shortage of autologous seed cells. Allogeneic or contralateral LESC-based therapies are constrained by demanding in vitro culture conditions, difficulties in scaling up, and risks of immune rejection, while MSCs and iPSCs offer a more abundant cell source. However, issues of immunogenicity and long-term stability persist. A recent application of iPSC-derived corneal epithelial sheets in four patients with LSCD showed promising therapeutic outcomes over a two-year observation period without serious adverse events. Future efforts should focus on expanding patient cohorts and conducting comparative studies with other allogeneic treatments for bilateral LSCD. EVs represent a promising strategy to circumvent immunogenicity. However, clinical translation is currently limited by challenges in isolation, purification, and standardized production. Harvesting methods such as ultracentrifugation are time-consuming, yield low quantities, and produce impurities. There is a pressing need to develop novel isolation techniques, such as microfluidics, immunoaffinity capture, size-exclusion chromatography, and membrane filtration, to achieve high-throughput, high-purity, and high-recovery EV production. Furthermore, establishing good manufacturing practice (GMP)-compliant processes is essential to ensure batch-to-batch consistency and stability. Strict quality control standards must be implemented to regulate physical properties, biochemical composition, bioactivity, sterility, endotoxin levels, and non-tumorigenicity of EVs. To address the short in vivo half-life of EVs, 4D printing could be harnessed to develop smart, stimuli-responsive biomaterials that enable controlled release and targeted delivery. Additionally, genetic engineering tools like CRISPR-Cas9 could be employed to enhance the proliferative and differentiation capacity of seed cells as well as to endow them with therapeutic functions, such as anti-inflammatory and anti-fibrotic properties, offering a novel source of engineered cells for transplantation.

Corneal epithelial organoids, generated through integrated innovative culture strategies, represent a highly promising alternative as corneal epithelial substitutes. By leveraging 3D organoid culture systems, the native microenvironment of CECs can be better mimicked, effectively preserving cell stemness and promoting proliferation. Moreover, organoids provide physiologically relevant spatial architectures and cell–cell interactions, which improve the distribution of nutrients and oxygen, thereby delaying cellular senescence and enhancing metabolic activity. Furthermore, by incorporating tailored matrix materials, precise growth factor modulation, and dynamic culture systems, organoids can yield CEC populations with higher viability and stability in vitro. It is also essential to develop and optimize serum-free or chemically defined culture media to reduce batch-to-batch variability, enhance cellular purity and safety, and minimize potential immunogenic risks.

In the development of biological scaffolds, enhancing bioactivity centers on the faithful mimicry of the native corneal microenvironment. Techniques such as electrospinning and bioprinting offer exceptional structural tunability and precision, enabling the creation of micro- and nanoscale topological features that guide cell alignment and tissue organization. A critical consideration remains the optimal blending of natural and synthetic materials to balance biocompatibility and mechanical strength. Bioprinting stands as one of the most pivotal manufacturing technologies. Its potential can be further amplified by refining printing parameters to minimize cell damage. Promising strategies include adopting 4D printing principles, incorporating nutrients and growth factors into bioinks, selecting larger nozzle diameters, and meticulously optimizing layer thickness and printing paths to enhance cell viability and functional integration. Cell sheets offer a compelling alternative by minimizing heterology and recreating natural ECM environments. However, its current limitations include poor mechanical strength and a lack of standardized manufacturing protocols. Future efforts should focus on optimizing culture conditions to improve mechanical properties and facilitate scalable production for clinical translation.

Bioengineering is advancing toward precision and personalized medicine, aiming to develop tailored corneal substitutes through the integration of cutting-edge technologies. High-resolution medical imaging techniques, such as optical coherence tomography and corneal topography, provide real-time, detailed cross-sectional images of damaged corneas, enabling continuous monitoring of scaffold integration during follow-up. Using computer-aided design, 3D digital models of patients’ corneal defects can be constructed. Finite element analysis then predicts the biomechanical behavior of implants under physiological stresses, allowing for pre-manufacturing assessment to ensure long-term stability. Subsequently, bioprinting technologies can be employed to fabricate complex, 3D corneal substitutes with high biocompatibility, thereby achieving precise patient-specific matching and maximizing the recovery of structural integrity and function. To further enhance production efficiency, machine learning (ML) algorithms are introduced. ML can analyze large volumes of experimental data to intelligently optimize bioprinting parameters, leading to more efficient and reproducible bioprinting processes while significantly reducing trial-and-error costs. By integrating multiomics data and cellular behavior profiles, ML models have the ability to predict cell behaviors within specific scaffolds and microenvironments. For instance, adhesion and spreading characteristics can be analyzed to forecast cell growth on different material surfaces, while the expression of specific genes may indicate the efficiency of differentiation into CECs. This enables the in vitro screening of optimal cell–scaffold combinations and supports the prediction of post-implantation outcomes, such as tissue transparency, biomechanical strength, and long-term stability. The integration of multiomics data into ML models offers a holistic understanding of the biological mechanisms underpinning corneal regeneration and provides deeper insights for personalized treatment strategies. Looking forward, the development of digital twin models of patient corneas could enable the simulation of various treatment outcomes in a virtual environment, thereby offering clinicians improved decision-making support and enabling predictions of long-term implant performance and potential risks.

## Figures and Tables

**Table 1:** Tissue engineering therapy for corneal epithelium regeneration. CLAU, conjunctival-limbal autograft; LSCD, limbal stem cell deficiency; CLET, cultivated limbal epithelial transplantation; SLET, simple limbal epithelial transplantation; CALEC, cultivated autologous limbal epithelial cell; COMET, cultured oral mucosal epithelial transplantation; MSC, mesenchymal stem cell; iPSC, induced pluripotent stem cell; CEC, corneal epithelial cell.

Therapy	Cell Resource	Description	Advantages	Disadvantages	Translation
CLAU	Autologous conjunctiva and corneal limbal tissue	Autologous transplantation of a large limbal tissue biopsy containing stem cells from the healthy contralateral eye to the affected eye	Simple to operate, high success rate	Suitable for unilateral LSCD, may induce iatrogenic LSCD in the donor eye, corneal conjunctivalization	Clinically conventional application
CLET	Autologous corneal limbal tissue	Harvesting a small amount of limbal tissue and expanding ex vivo on scaffolds to form epithelial cell sheets	Suitable for bilateral LSCD, less traumatic	Complicated technique, high cost	Holoclar
SLET	Autologous/allogeneic corneal limbal tissue	Harvesting a small amount of limbal tissue from a healthy contralateral eye, which is divided into micrografts and directly adhered to an amniotic membrane carrier	Less traumatic, no need for ex vivo culture, simple to operate, high success rate	Suitable for unilateral LSCD, need for long-term immunosuppression	Clinically conventional application clinically
CALEC	Autologous corneal limbal tissue	A standardized CLET technique utilizing a xeno-free culture system	Xeno-free, production reproducibility	Complicated technique, the highest cost	Phase II completes
COMET	Autologous oral mucosal epithelium	Harvesting a small amount of oral mucosal epithelium tissue and expanding ex vivo on scaffolds to form epithelial cell sheets	Suitable for bilateral LSCD, convenient material selection, adequate sources	Morphological and functional mismatch with corneal epithelium, poor visual outcome	Ocural^®^, Sakracy^®^
MSC therapy	Allogeneic MSC	Allogeneic MSC-induced CEC with bioscaffolds	Suitable for bilateral LSCD, adequate sources	Complicated technique, immunosuppression	Preclinical study
iPSC therapy	Autologous somatic cell	Reprogrammed autologous somatic cell to CEC with bioscaffolds	Adequate source, free of immunosuppression	Extremely complicated technique, high cost, tumorigenicity	Preclinical study
Cell sheet	Autologous/Allogeneic CEC	Formed primarily through self-assembled cell-secreted ECM	Closest to a natural structure, free of external materials	Complicated technique, poor mechanical strength	Nepic^®^

**Table 2:** Summary of extrusion 3D printing bioinks and diameters of cornea tissue engineering. GelMA, gelatin methacrylate; PEGDA, long-chain poly (ethylene glycol) diacrylate; CEC, corneal epithelium cell; ASC, adipose-derived mesenchymal stem cell; DCM, decellularized cornea matrix; SF, silk fibroin; LMSC, limbal mesenchymal stem cell; ColMA, methacrylated collagen; HA-CDH, conjugation of carbodihydrazide on hyaluronic acid; HA-Ald, carbodihydrazide-aldehyde; iPSC-CEnC, iPSC-induced corneal endothelium cell.

Bioink	Nozzle Diameters	Printing Temperature	Fixation	Cell Viability	Reference
GelMA, keratocyte	Not mentioned	20 °C	UV	≈80%	[155]
PEGDA + GelMA, CEC + ASC	Not mentioned	37 °C	Blue light	90%	[156]
EPTAC-Col + GelMA, keratocyte	0.34 mm	20 °C	UV	95%	[157]
DCM + SF, LMSC	0.33 mm	15 °C	Green light	92%	[158]
Fibrin, LESC	0.2 mm	Not mentioned	CaCl_2_	91.1%	[159]
Sodium alginate +ColMA, keratocytes	0.2 mm	37 °C	CaCl_2_	83%	[160]
GelMA, keratocytes	0.26 mm	Not mentioned	UV	98%	[161]
HA-CDH + HA-Ald, ASC + keratocyte	0.1 mm	20 °C	Hydrazone	95%	[162]
HA-CDH + HA-Ald, iPSC-CEnC	0.1 mm	Room temperature	Hydrazone	92.5%	[163]

## Data Availability

The original contributions presented in the study are included in the article, further inquiries can be directed to the corresponding author.

## Abbreviations

The following abbreviations are used in this manuscript:

LESC	Limbal epithelial stem cell
LSCD	Limbal stem cell deficiency
ECM	Extracellular matrix
CEC	Corneal epithelial cell
3D	Three-dimensional
MSC	Mesenchymal stem cell
iPSC	Induced pluripotent stem cell
COMET	Cultured mucosal epithelial transplantation
CLAU	Conjunctival–limbal autograft
KLAL	Keratolimbal allograft
CLET	Cultivated limbal epithelial transplantation
SLET	Simple limbal epithelial transplantation
CALEC	Cultivated autologous limbal epithelial cell
hOMSC	Human oral mucosa stem cell
ESC	Embryonic stem cell
CHC	Carboxymethyl-hexanoyl chitosan
SEAM	Self-formed ectoderm autonomous multizone
EV	Extracellular vesicle
ALI	Air–liquid interface
SAW	Surface acoustic wave
ASC	Adipose-derived mesenchymal stem cell
DCM	Decellularized cornea matrix
LMSC	Limbal mesenchymal stem cell
ML	Machine learning

## References

[1] Barrientez B., Nicholas S.E., Whelchel A., Sharif R., Hjortdal J., Karamichos D. (2019). Corneal injury: Clinical and molecular aspects. Exp. Eye Res..

[2] Mohan R.R., Kempuraj D., D’Souza S., Ghosh A. (2022). Corneal stromal repair and regeneration. Prog. Retin. Eye Res..

[3] Wang E.Y., Kong X., Wolle M., Gasquet N., Ssekasanvu J., Mariotti S.P., Bourne R., Taylor H., Resnikoff S., West S. (2023). Global Trends in Blindness and Vision Impairment Resulting from Corneal Opacity 1984–2020: A Meta-analysis. Ophthalmology.

[4] Whitcher J.P., Srinivasan M., Upadhyay M.P. (2001). Corneal blindness: A global perspective. Bull. World Health Organ..

[5] Tran T.M., Duong H., Bonnet C., Kashanchi A., Buckshey A., Aldave A.J. (2020). Corneal Blindness in Asia: A Systematic Review and Meta-Analysis to Identify Challenges and Opportunities. Cornea.

[6] Wu D.H., Lin Y., Wu H.P., Cai J.H. (2025). Trauma-induced corneal epithelial defects may lead to persistent epithelial defects exacerbated by prolonged use of bandage lenses. Eur. J. Ophthalmol..

[7] Rodriguez-Garcia A., Ruiz-Lozano R.E., Barcelo-Canton R.H., Marines-Sanchez H.M., Homar Paez-Garza J. (2025). The etiologic and pathogenic spectrum of exposure keratopathy: Diagnostic and therapeutic implications. Surv. Ophthalmol..

[8] Georgoudis P. (2024). Persistent epithelial defects in neurotrophic keratopathy. Acta Ophthalmol..

[9] Moussa S.M., Mahmoud S.S., Aly E.M., Talaat M.S. (2024). Bio-spectroscopic analysis of corneal structural alterations in dry eye disease: A study of collagen, co-enzymes, lipids, and proteins with emphasis on phytotherapy intervention. Int. J. Biol. Macromol..

[10] Ladea L., Zemba M., Calancea M.I., Caltaru M.V., Dragosloveanu C.D.M., Coroleuca R., Catrina E.L., Brezean I., Dinu V. (2024). Corneal Epithelial Changes in Diabetic Patients: A Review. Int. J. Mol. Sci..

[11] Anju M.S., Mathew A.I., Raj D.K., Vinod D., Kasoju N., Raghavan C., Kumar P.R.A. (2025). Bioengineered Human Limbal Stem Cell-Derived Epithelial Sheets for Ocular Surface Reconstruction. Regen. Eng. Transl. Med..

[12] Lazzara F., Conti F., Maugeri G., D’Agata V., Sotera L., Bucolo C. (2025). Corneal protective effects of a new ophthalmic formulation based on vitamin B12 and sodium hyaluronate. Front. Pharmacol..

[13] Gelles J.D., Hillier K.E., Krisa S., Greenstein S.A., Hersh P.S. (2022). Lipid Keratopathy Management with Therapeutic Scleral Lens Wear. Eye Contact Lens.

[14] Moon J., Lee S.M., Hyon J.Y., Kim M.K., Oh J.Y., Choi H.J. (2021). Large diameter scleral lens benefits for Asians with intractable ocular surface diseases: A prospective, single-arm clinical trial. Sci. Rep..

[15] Dikmetas O., Kapucu Y., Cankaya A.B., Kocabeyoglu S. (2024). Outcomes and success of amniotic membrane transplantation for the treatment of corneal diseases. Cutan. Ocul. Toxicol..

[16] Schuerch K., Baeriswyl A., Frueh B.E., Tappeiner C. (2020). Efficacy of Amniotic Membrane Transplantation for the Treatment of Corneal Ulcers. Cornea.

[17] Tsai R.J., Li L.M., Chen J.K. (2000). Reconstruction of damaged corneas by transplantation of autologous limbal epithelial cells. N. Engl. J. Med..

[18] Nguyen K.N., Bobba S., Richardson A., Park M., Watson S.L., Wakefield D., Di Girolamo N. (2018). Native and synthetic scaffolds for limbal epithelial stem cell transplantation. Acta Biomater..

[19] Alio J.L., Montesel A., El Sayyad F., Barraquer R.I., Arnalich-Montiel F. (2021). Corneal graft failure: An update. Br. J. Ophthalmol..

[20] Kumar R., Sinha N.R., Mohan R.R. (2023). Corneal gene therapy: Structural and mechanistic understanding. Ocul. Surf..

[21] Li S., Sun H., Chen L., Fu Y. (2024). Targeting limbal epithelial stem cells: Master conductors of corneal epithelial regeneration from the bench to multilevel theranostics. J. Transl. Med..

[22] Nosrati H., Alizadeh Z., Nosrati A., Ashrafi-Dehkordi K., Banitalebi-Dehkordi M., Sanami S., Khodaei M. (2021). Stem cell-based therapeutic strategies for corneal epithelium regeneration. Tissue Cell.

[23] Bisevac J., Katta K., Petrovski G., Moe M.C., Noer A. (2023). Wnt/β-Catenin Signaling Activation Induces Differentiation in Human Limbal Epithelial Stem Cells Cultured Ex Vivo. Biomedicines.

[24] Shafiq M., Ali O., Han S.B., Kim D.H. (2021). Mechanobiological Strategies to Enhance Stem Cell Functionality for Regenerative Medicine and Tissue Engineering. Front. Cell Dev. Biol..

[25] Ricciotti L., Apicella A., Perrotta V., Aversa R. (2023). Geopolymer Materials for Bone Tissue Applications: Recent Advances and Future Perspectives. Polymers.

[26] Xu H.Q., Liu J.C., Zhang Z.Y., Xu C.X. (2022). A review on cell damage, viability, and functionality during 3D bioprinting. Mil. Med. Res..

[27] Gouveia R.M., Vajda F., Wibowo J.A., Figueiredo F., Connon C.J. (2019). YAP, ΔNp63, and β-Catenin Signaling Pathways Are Involved in the Modulation of Corneal Epithelial Stem Cell Phenotype Induced by Substrate Stiffness. Cells.

[28] Huang X., Huang Z.X., Gao W.D., He R.Y., Li Y.L., Crawford R., Zhou Y.H., Xiao L., Xiao Y. (2022). Current Advances in 3D Dynamic Cell Culture Systems. Gels.

[29] Eghrari A.O., Riazuddin S.A., Gottsch J.D. (2015). Overview of the Cornea: Structure, Function, and Development. Prog. Mol. Biol. Transl. Sci..

[30] Chirila T.V., Hicks C.R., Dalton P.D., Vijayasekaran S., Lou X., Hong Y., Clayton A.B., Ziegelaar B.W., Fitton J.H., Platten S. (1998). Artificial cornea. Prog. Polym. Sci..

[31] Steinhoff G. (2016). Regenerative Medicine—From Protocol to Patient: 1. Biology of Tissue Regeneration.

[32] Gonzalez G., Sasamoto Y., Ksander B.R., Frank M.H., Frank N.Y. (2018). Limbal stem cells: Identity, developmental origin, and therapeutic potential. Wiley Interdiscip. Rev. Dev. Biol..

[33] Hanna C., Bicknell D.S., O’Brien J.E. (1961). Cell turnover in the adult human eye. Arch. Ophthalmol..

[34] Cotsarelis G., Cheng S.Z., Dong G., Sun T.T., Lavker R.M. (1989). Existence of slow-cycling limbal epithelial basal cells that can be preferentially stimulated to proliferate: Implications on epithelial stem cells. Cell.

[35] Sartaj R., Zhang C., Wan P., Pasha Z., Guaiquil V., Liu A., Liu J., Luo Y., Fuchs E., Rosenblatt M.I. (2017). Characterization of slow cycling corneal limbal epithelial cells identifies putative stem cell markers. Sci. Rep..

[36] Altshuler A., Amitai-Lange A., Tarazi N., Dey S., Strinkovsky L., Hadad-Porat S., Bhattacharya S., Nasser W., Imeri J., Ben-David G. (2021). Discrete limbal epithelial stem cell populations mediate corneal homeostasis and wound healing. Cell Stem Cell.

[37] Kuo W.T., Odenwald M.A., Turner J.R., Zuo L. (2022). Tight junction proteins occludin and ZO-1 as regulators of epithelial proliferation and survival. Ann. N. Y. Acad. Sci..

[38] Hou A.H., Ali S.M., Png E., Hunziker W., Tong L.I. (2023). Transglutaminase-2 is critical for corneal epithelial barrier function via positive regulation of Claudin-1. Ocul. Surf..

[39] Giasson C.J., Deschambeault A., Carrier P., Germain L. (2014). Adherens junction proteins are expressed in collagen corneal equivalents produced in vitro with human cells. Mol. Vis..

[40] Bush J., Cabe J.I., Conway D., Maruthamuthu V. (2023). E-cadherin adhesion dynamics as revealed by an accelerated force ramp are dependent upon the presence of α-catenin. Biochem. Biophys. Res. Commun..

[41] Shen S., Zhang Y. (2024). Restoration of corneal epithelial barrier function: A possible target for corneal neovascularization. Ocul. Surf..

[42] Ebrahim A.S., Carion T.W., Ebrahim T., Win J., Kani H., Wang Y.X., Stambersky A., Ibrahim A.S., Sosne G., Berger E.A. (2023). A Novel Combination Therapy Tβ4/VIP Protects against Hyperglycemia-Induced Changes in Human Corneal Epithelial Cells. Biosensors.

[43] Wang L.Y., Xu X.Z., Chen Q.K., Wei Y., Wei Z.Y., Jin Z.B., Liang Q.F. (2023). Extracellular Vesicle MicroRNAs from Corneal Stromal Stem Cell Enhance Stemness of Limbal Epithelial Stem Cells by Targeting the Notch Pathway. Investig. Ophthalmol. Vis. Sci..

[44] Plamann K. (2024). Optics and quantitative assessment of corneal transparency. Acta Ophthalmol..

[45] Maurice D.M. (1957). The Structure and Transparency of the Cornea. J. Physiol..

[46] Sang S.B., Yan Y.Y., Shen Z.Z., Cao Y.Y., Duan Q.Q., He M., Zhang Q. (2022). Photo-crosslinked hydrogels for tissue engineering of corneal epithelium. Exp. Eye Res..

[47] Cen Y.J., You D.B., Wang W., Feng Y. (2021). Preliminary studies of constructing a tissue-engineered lamellar corneal graft by culturing mesenchymal stem cells onto decellularized corneal matrix. Int. J. Ophthalmol..

[48] Visalli F., Fava F., Capobianco M., Musa M., D’Esposito F., Russo A., Scollo D., Longo A., Gagliano C., Zeppieri M. (2024). Innovative Bioscaffolds in Stem Cell and Regenerative Therapies for Corneal Pathologies. Bioengineering.

[49] Kato D., Hirano K., Tanikawa A., Ito Y. (2025). Corneal surface reconstruction for the chemical injured eye by transplanting autologous cultivated limbal epithelial sheet “Nepic^®^”. Fujita Med. J..

[50] Nakamura T., Kinoshita S. (2003). Ocular surface reconstruction using cultivated mucosal epithelial stem cells. Cornea.

[51] Nakamura T., Inatomi T., Sotozono C., Amemiya T., Kanamura N., Kinoshita S. (2004). Transplantation of cultivated autologous oral mucosal epithelial cells in patients with severe ocular surface disorders. Br. J. Ophthalmol..

[52] Nishida K., Yamato M., Hayashida Y., Watanabe K., Yamamoto K., Adachi E., Nagai S., Kikuchi A., Maeda N., Watanabe H. (2004). Corneal reconstruction with tissue-engineered cell sheets composed of autologous oral mucosal epithelium. N. Engl. J. Med..

[53] Toshida H., Kasahara T., Kiriyama M., Iwasaki Y., Sugita J., Ichikawa K., Ohta T., Miyahara K. (2023). Early Clinical Outcomes of the First Commercialized Human Autologous Ex Vivo Cultivated Oral Mucosal Epithelial Cell Transplantation for Limbal Stem Cell Deficiency: Two Case Reports and Literature Review. Int. J. Mol. Sci..

[54] Kimura K., Imai K., Ueno M., Sotozono C. (2025). Assessment of the official national insurance coverage of regenerative medical products for ophthalmic diseases in Japan following regulatory approval. Regen. Ther..

[55] Le Q.H., Chauhan T., Yung M., Tseng C.H., Deng S.X. (2020). Outcomes of Limbal Stem Cell Transplant A Meta-analysis. JAMA Ophthalmol..

[56] Daya S.M. (2017). Conjunctival-limbal autograft. Curr. Opin. Ophthalmol..

[57] Eslani M., Cheung A.Y., Kurji K., Pierson K., Sarnicola E., Holland E.J. (2019). Long-term outcomes of conjunctival limbal autograft in patients with unilateral total limbal stem cell deficiency. Ocul. Surf..

[58] Kenyon K.R., Tseng S.C.G. (1989). Limbal Autograft Transplantation for Ocular Surface Disorders. Ophthalmology.

[59] Chan C.C., Biber J.M., Holland E.J. (2012). The Modified Cincinnati Procedure: Combined Conjunctival Limbal Autografts and Keratolimbal Allografts for Severe Unilateral Ocular Surface Failure. Cornea.

[60] Cheung A.Y., Eslani M., Kurji K.H., Wright E., Sarnicola E., Govil A., Holland E.J. (2020). Long-term Outcomes of Living-Related Conjunctival Limbal Allograft Compared with Keratolimbal Allograft in Patients with Limbal Stem Cell Deficiency. Cornea.

[61] Pellegrini G., Traverso C.E., Franzi A.T., Zingirian M., Cancedda R., DeLuca M. (1997). Long-term restoration of damaged corneal surfaces with autologous cultivated corneal epithelium. Lancet.

[62] Liu J.B., Lawrence B.D., Liu A.H., Schwab I.R., Oliveira L.A., Rosenblatt M.I. (2012). Silk Fibroin as a Biomaterial Substrate for Corneal Epithelial Cell Sheet Generation. Investig. Ophthalmol. Vis. Sci..

[63] Schwab I.R., Reyes M., Isseroff R.R. (2000). Successful transplantation of bioengineered tissue replacements in patients with ocular surface disease. Cornea.

[64] Ghareeb A.E., Lako M., Figueiredo F.C. (2020). Recent Advances in Stem Cell Therapy for Limbal Stem Cell Deficiency: A Narrative Review. Ophthalmol. Ther..

[65] Pellegrini G., Ardigò D., Milazzo G., Iotti G., Guatelli P., Pelosi D., De Luca M. (2018). Navigating Market Authorization: The Path Holoclar Took to Become the First Stem Cell Product Approved in the European Union. Stem Cell Transl. Med..

[66] Sangwan V.S., Basu S., MacNeil S., Balasubramanian D. (2012). Simple limbal epithelial transplantation (SLET): A novel surgical technique for the treatment of unilateral limbal stem cell deficiency. Br. J. Ophthalmol..

[67] Jurkunas U., Johns L., Armant M. (2022). Cultivated Autologous Limbal Epithelial Cell Transplantation: New Frontier in the Treatment of Limbal Stem Cell Deficiency. Am. J. Ophthalmol..

[68] Jurkunas U.V., Kaufman A.R., Yin J., Ayala A., Maguire M., Samarakoon L., Johns L.K., Parekh M., Li S., Gauthier A. (2025). Cultivated autologous limbal epithelial cell (CALEC) transplantation for limbal tem cell deficiency: A phase I/II clinical trial of the first xenobiotic-free, serum-free, antibiotic-free manufacturing protocol developed in the US. Nat. Commun..

[69] Kacham S., Bhure T.S., Eswaramoorthy S.D., Naik G., Rath S.N., Parcha S.R., Basu S., Sangwan V.S., Shukla S. (2021). Human Umbilical Cord-Derived Mesenchymal Stem Cells Promote Corneal Epithelial Repair In Vitro. Cells.

[70] Long Q.R., Huang C., Zhang L.Y., Jiang H., Zhao S., Zhang L.L., Zheng X.E., Ou S.K., Gu H. (2024). A novel tissue-engineered corneal epithelium based on ultra-thin amniotic membrane and mesenchymal stem cells. Sci. Rep..

[71] Kobal N., Marzidovsek M., Schollmayer P., Malicev E., Hawlina M., Marzidovsek Z.L. (2024). Molecular and Cellular Mechanisms of the Therapeutic Effect of Mesenchymal Stem Cells and Extracellular Vesicles in Corneal Regeneration. Int. J. Mol. Sci..

[72] Yao L., Li Z.R., Su W.R., Li Y.P., Lin M.L., Zhang W.X., Liu Y., Wan Q., Liang D. (2012). Role of Mesenchymal Stem Cells on Cornea Wound Healing Induced by Acute Alkali Burn. PLoS ONE.

[73] Fu Y.O., Chen P.R., Yeh C.C., Pan J.Y., Kuo W.C., Tseng K.W. (2022). Human Umbilical Mesenchymal Stem Cell Xenografts Repair UV-Induced Photokeratitis in a Rat Model. Biomedicines.

[74] Venkatakrishnan J., Saeed Y., Kao W.W.Y. (2022). Trends in using mesenchymal stromal/stem cells (MSCs) in treating corneal diseases. Ocul. Surf..

[75] Marynka-Kalmani K., Treves S., Yafee M., Rachima H., Gafni Y., Cohen M.A., Pitaru S. (2010). The Lamina Propria of Adult Human Oral Mucosa Harbors a Novel Stem Cell Population. Stem Cells.

[76] López S., Hoz L., Tenorio E.P., Buentello B., Magaña F.S., Wintergerst A., Navas A., Garfias Y., Arzate H. (2021). Can Human Oral Mucosa Stem Cells Differentiate to Corneal Epithelia?. Int. J. Mol. Sci..

[77] Chien Y., Liao Y.W., Liu D.M., Lin H.L., Chen S.J., Chen H.L., Peng C.H., Liang C.M., Mou C.Y., Chiou S.H. (2012). Corneal repair by human corneal keratocyte-reprogrammed iPSCs and amphiphatic carboxymethyl-hexanoyl chitosan hydrogel. Biomaterials.

[78] Yu D., Chen M.F., Sun X.R., Ge J. (2013). Differentiation of mouse induced pluripotent stem cells into corneal epithelial-like cells. Cell Biol. Int..

[79] Lee S., Han J., Yang J., Lyu J., Park H., Bang J., Kim Y., Chang H., Park T. (2024). Exosomes from Human iPSC-Derived Retinal Organoids Enhance Corneal Epithelial Wound Healing. Int. J. Mol. Sci..

[80] Hayashi R., Ishikawa Y., Sasamoto Y., Katori R., Nomura N., Ichikawa T., Araki S., Soma T., Kawasaki S., Sekiguchi K. (2016). Co-ordinated ocular development from human iPS cells and recovery of corneal function. Nature.

[81] Soma T., Oie Y., Takayanagi H., Matsubara S., Yamada T., Nomura M., Yoshinaga Y., Maruyama K., Watanabe A., Takashima K. (2024). Induced pluripotent stem-cell-derived corneal epithelium for transplant surgery: A single-arm, open-label, first-in-human interventional study in Japan. Lancet.

[82] Tan F., Li X.R., Wang Z., Li J.J., Shahzad K., Zheng J.L. (2024). Clinical applications of stem cell-derived exosomes. Signal Transduct. Target. Ther..

[83] Samaeekia R., Rabiee B., Putra I., Shen X., Park Y.J., Hematti P., Eslani M., Djalilian A.R. (2018). Effect of Human Corneal Mesenchymal Stromal Cell-derived Exosomes on Corneal Epithelial Wound Healing. Investig. Ophthalmol. Vis. Sci..

[84] Tang Q.M., Lu B., He J., Chen X., Fu Q.L., Han H.J., Luo C.Q., Yin H.F., Qin Z.W., Lyu D.N. (2022). Exosomes-loaded thermosensitive hydrogels for corneal epithelium and stroma regeneration. Biomaterials.

[85] Sun X.M., Song W.J., Teng L.J., Huang Y.R., Liu J., Peng Y.H., Lu X.T., Yuan J., Zhao X., Zhao Q. (2023). MiRNA 24-3p-rich exosomes functionalized DEGMA-modified hyaluronic acid hydrogels for corneal epithelial healing. Bioact. Mater..

[86] Xu Y.H., Wei C., Ma L., Zhao L., Li D.F., Lin Y.L., Zhou Q.J., Xie L.X. (2025). 3D mesenchymal stem cell exosome-functionalized hydrogels for corneal wound healing. J. Control. Release.

[87] Wang H., Alarcon C.N., Liu B., Watson F., Searles S., Lee C.K., Keys J., Pi W., Allen D., Lammerding J. (2022). Genetically engineered and enucleated human mesenchymal stromal cells for the targeted delivery of therapeutics to diseased tissue. Nat. Biomed. Eng..

[88] Hampel U., Garreis F., Burgemeister F., Essel N., Paulsen F. (2018). Effect of intermittent shear stress on corneal epithelial cells using an flow culture model. Ocul. Surf..

[89] Molladavoodi S., Robichaud M., Wulff D., Gorbet M. (2017). Corneal epithelial cells exposed to shear stress show altered cytoskeleton and migratory behaviour. PLoS ONE.

[90] Utsunomiya T., Ishibazawa A., Yoshioka T., Song Y.S., Yoshida K. (2023). Assessing effects of mechanical stimulation of fluid shear stress on inducing matrix Metalloproteinase-9 in cultured corneal epithelial cells. Exp. Eye Res..

[91] Wu M.F., Peng X., Zhao J.L., Zhang M.C., Xie H.T. (2023). Mitophagy and mitochondrion-related expression profiles in response to physiological and pathological hypoxia in the corneal epithelium. Genomics.

[92] Ma X.L., Liu H.Q. (2011). Effect of hypoxia on the proliferation of murine cornea limbal epithelial progenitor cells. Int. J. Ophthalmol..

[93] Fehrer C., Brunauer R., Laschober G., Unterluggauer H., Reitinger S., Kloss F., Gülly C., Gassner R., Lepperdinger G. (2007). Reduced oxygen tension attenuates differentiation capacity of human mesenchymal stem cells and prolongs their lifespan. Aging Cell.

[94] Han K.H., Kim A.K., Kim M.H., Kim D.H., Go H.N., Kim D.I. (2016). Enhancement of angiogenic effects by hypoxia-preconditioned human umbilical cord-derived mesenchymal stem cells in a mouse model of hindlimb ischemia. Cell Biol. Int..

[95] Kaitsuka T., Hakim F. (2021). Response of Pluripotent Stem Cells to Environmental Stress and Its Application for Directed Differentiation. Biology.

[96] Wu J.X., Yu L., Liu Y., Xiao B., Ye X.J., Zhao H., Xi Y.H., Shi Z.C., Wang W.H. (2023). Hypoxia regulates adipose mesenchymal stem cells proliferation, migration, and nucleus pulposus-like differentiation by regulating endoplasmic reticulum stress via the HIF-1α pathway. J. Orthop. Surg. Res..

[97] Wang Y.W., Xu L.L., Zhao J.P., Liang J.C., Zhang Z.X., Li Q., Zhang J.H., Wan P.X., Wu Z. (2022). Reconstructing auto tissue engineering lamellar cornea with aspartic acid modified acellular porcine corneal stroma and preconditioned limbal stem cell for corneal regeneration. Biomaterials.

[98] Seo Y., Shin T.H., Kim H.S. (2019). Current Strategies to Enhance Adipose Stem Cell Function: An Update. Int. J. Mol. Sci..

[99] Takeuchi S., Tsuchiya A., Iwasawa T., Nojiri S., Watanabe T., Ogawa M., Yoshida T., Fujiki K., Koui Y., Kido T. (2021). Small extracellular vesicles derived from interferon-γ pre-conditioned mesenchymal stromal cells effectively treat liver fibrosis. npj Regen. Med..

[100] Nakao Y., Fukuda T., Zhang Q.Z., Sanui T., Shinjo T., Kou X.X., Chen C., Liu D.W., Watanabe Y., Hayashi C. (2021). Exosomes from TNF-α-treated human gingiva-derived MSCs enhance M2 macrophage polarization and inhibit periodontal bone loss. Acta Biomater..

[101] Heo S.C., Jeon E.S., Lee I.H., Kim H.S., Kim M.B., Kim J.H. (2011). Tumor Necrosis Factor-α-Activated Human Adipose Tissue-Derived Mesenchymal Stem Cells Accelerate Cutaneous Wound Healing through Paracrine Mechanisms. J. Investig. Dermatol..

[102] Abuwatfa W.H., Pitt W.G., Husseini G.A. (2024). Scaffold-based 3D cell culture models in cancer research. J. Biomed. Sci..

[103] Monostori T., Szucs D., Lovászi B., Kemény L., Veréb Z. (2024). Advances in tissue engineering and 3D bioprinting for corneal regeneration. Int. J. Bioprint..

[104] Zhang M., Yang F., Han D., Zhang S.Y., Dong Y., Li X., Ling L., Deng Z., Cao X., Tian J. (2023). 3D bioprinting of corneal decellularized extracellular matrix: GelMA composite hydrogel for corneal stroma engineering. Int. J. Bioprint..

[105] Lekkala V.K.R., Kang S.Y., Liu J., Shrestha S., Acharya P., Joshi P., Zolfaghar M., Lee M., Vanga M.G., Jamdagneya P. (2024). A Pillar/Perfusion Plate Enhances Cell Growth, Reproducibility, Throughput, and User Friendliness in Dynamic 3D Cell Culture. ACS Biomater. Sci. Eng..

[106] Abdalkader R., Kamei K.I. (2020). Multi-corneal barrier-on-a-chip to recapitulate eye blinking shear stress forces. Lab Chip.

[107] Bennet D., Estlack Z., Reid T., Kim J. (2018). A microengineered human corneal epithelium-on-a-chip for eye drops mass transport evaluation. Lab Chip.

[108] Lee R., Kim H., Kim H., Lee J., Cho K.J., Kim J. (2023). High throughput microfluidic drug screening system for corneal epithelial wound healing. J. Micromech. Microeng..

[109] Wu Z., Zhou Q., Duan H.Y., Wang X.R., Xiao J.H., Duan H.C., Li N.Y., Li C.Y., Wan P.X., Liu Y. (2014). Reconstruction of Auto-Tissue-Engineered Lamellar Cornea by Dynamic Culture for Transplantation: A Rabbit Model. PLoS ONE.

[110] Greco G., Agostini M., Tonazzin I., Sallemi D., Barone S., Cecchini M. (2018). Surface-Acoustic-Wave (SAW)-Driven Device for Dynamic Cell Cultures. Anal. Chem..

[111] Gao Y., Fajrial A.K., Yang T., Ding X.Y. (2021). Emerging on-chip surface acoustic wave technology for small biomaterials manipulation and characterization. Biomater. Sci..

[112] Gosselin E.A., Torregrosa T., Ghezzi C.E., Mendelsohn A.C., Gomes R., Funderburgh J.L., Kaplan D.L. (2018). Multi-layered silk film coculture system for human corneal epithelial and stromal stem cells. J. Tissue Eng. Regen. Med..

[113] An S., Anwar K., Ashraf M., Lee H., Jung R.B.C., Koganti R., Ghassemi M., Djalilian A.R. (2023). Wound-Healing Effects of Mesenchymal Stromal Cell Secretome in the Cornea and the Role of Exosomes. Pharmaceutics.

[114] Shen T., Shen J., Zheng Q.Q., Li Q.S., Zhao H.L., Cui L., Hong C.Y. (2017). Cell viability and extracellular matrix synthesis in a co-culture system of corneal stromal cells and adipose-derived mesenchymal stem cells. Int. J. Ophthalmol..

[115] Soleimanifar F., Mortazavi Y., Nadri S., Islami M., Vakilian S. (2018). Coculture of conjunctiva derived mesenchymal stem cells (CJMSCs) and corneal epithelial cells to reconstruct the corneal epithelium. Biologicals.

[116] Moreno I.Y., Parsaie A., Verma S., Gesteira T.F., Coulson-Thomas V.J. (2023). Characterization of the Limbal Epithelial Stem Cell Niche. Investig. Ophthalmol. Vis. Sci..

[117] Ahmadiankia N., Ebrahimi M., Hosseini A., Baharvand H. (2009). Effects of different extracellular matrices and co-cultures on human limbal stem cell expansion in vitro. Cell Biol. Int..

[118] Nam S.M., Maeng Y.S., Kim E.K., Seo K.Y., Lew H. (2017). Ex Vivo Expansion of Human Limbal Epithelial Cells Using Human Placenta-Derived and Umbilical Cord-Derived Mesenchymal Stem Cells. Stem Cells Int..

[119] Dziasko M.A., Tuft S.J., Daniels J.T. (2015). Limbal melanocytes support limbal epithelial stem cells in 2D and 3D microenvironments. Exp. Eye Res..

[120] Liu X.Y., Chen H., Zhou Q., Wu J., Zhang X.L., Wang L., Qin X.Y. (2013). Tissue engineering of lamellar cornea using human amniotic epithelial cells and rabbit cornea stroma. Int. J. Ophthalmol..

[121] Ajjarapu S.M., Tiwari A., Kumar S. (2023). Applications and Utility of Three-Dimensional In Vitro Cell Culture for Therapeutics. Future Pharmacol..

[122] Duval K., Grover H., Han L.H., Mou Y., Pegoraro A.F., Fredberg J., Chen Z. (2017). Modeling Physiological Events in 2D vs. 3D Cell Culture. Physiology.

[123] Carter K., Lee H.J., Na K.S., Fernandes-Cunha G.M., Blanco I.J., Djalilian A., Myung D. (2019). Characterizing the impact of 2D and 3D culture conditions on the therapeutic effects of human mesenchymal stem cell secretome on corneal wound healing in vitro and ex vivo. Acta Biomater..

[124] Sumbalova Koledova Z. (2024). 3D Cell Culture: Techniques For and Beyond Organoid Applications. Methods in Molecular Biology.

[125] Wu Z., Su X., Xu Y., Kong B., Sun W., Mi S. (2016). Bioprinting three-dimensional cell-laden tissue constructs with controllable degradation. Sci. Rep..

[126] Li S., Han Y., Lei H., Zeng Y., Cui Z., Zeng Q., Zhu D., Lian R., Zhang J., Chen Z. (2017). In vitro biomimetic platforms featuring a perfusion system and 3D spheroid culture promote the construction of tissue-engineered corneal endothelial layers. Sci. Rep..

[127] Higa K., Higuchi J., Kimoto R., Miyashita H., Shimazaki J., Tsubota K., Shimmura S. (2020). Human corneal limbal organoids maintaining limbal stem cell niche function. Stem Cell Res..

[128] Wilson S.E., Torricelli A.A.M., Marino G.K. (2020). Corneal epithelial basement membrane: Structure, function and regeneration. Exp. Eye Res..

[129] Smita S.S., Pramanik K. (2025). Fabrication and characterization of an electrospun silk fibroin/gelatin transparent graft material for corneal epithelial regeneration. Int. J. Polym. Mater. Polym. Biomater..

[130] Last J.A., Liliensiek S.J., Nealey P.F., Murphy C.J. (2009). Determining the mechanical properties of human corneal basement membranes with atomic force microscopy. J. Struct. Biol..

[131] Salimbeigi G., Vrana N.E., Ghaemmaghami A.M., Huri P.Y., McGuinness G.B. (2022). Basement membrane properties and their recapitulation in organ-on-chip applications. Mater. Today Bio.

[132] Sun M.G., Luo Y.C., Teng T., Guaiquil V., Zhou Q., McGinn L., Nazzal O., Walsh M., Lee J., Rosenblatt M.I. (2021). Silk Film Stiffness Modulates Corneal Epithelial Cell Mechanosignaling. Macromol. Chem. Phys..

[133] Lei H.Y., Yi T., Fan H.Y., Pei X., Wu L.N., Xing F., Li M.X., Liu L., Zhou C.C., Fan Y.J. (2021). Customized additive manufacturing of porous Ti6Al4V scaffold with micro-topological structures to regulate cell behavior in bone tissue engineering. Mater. Sci. Eng. C.

[134] Hu X., Bao M. (2024). Advances in micropatterning technology for mechanotransduction research. Mechanobiol. Med..

[135] Aboal-Castro L., Radziunas-Salinas Y., Pita-Vilar M., Carnero B., Mikos A.G., Alvarez-Lorenzo C., Flores-Arias M.T., Diaz-Gomez L. (2025). Laser-Assisted Micropatterned 3D Printed Scaffolds with Customizable Surface Topography and Porosity for Modulation of Cell Function. Adv. Healthc. Mater..

[136] Lawrence B.D., Pan Z., Liu A., Kaplan D.L., Rosenblatt M.I. (2012). Human corneal limbal epithelial cell response to varying silk film geometric topography in vitro. Acta Biomater..

[137] Kang K.B., Lawrence B.D., Gao X.R., Guaiquil V.H., Liu A., Rosenblatt M.I. (2019). The Effect of Micro- and Nanoscale Surface Topographies on Silk on Human Corneal Limbal Epithelial Cell Differentiation. Sci. Rep..

[138] Öncel M.Ö.Ö., Erkoc-Biradli F.Z., Rasier R., Marcali M., Elbuken C., Garipcan B. (2021). Rose petal topography mimicked poly(dimethylsiloxane) substrates for enhanced corneal endothelial cell behavior. Mat. Sci. Eng. C.

[139] Wang Q.S., Liu Q.S., Gao J.M., He J.H., Zhang H.J., Ding J.D. (2023). Stereo Coverage and Overall Stiffness of Biomaterial Arrays Underly Parts of Topography Effects on Cell Adhesion. ACS Appl. Mater. Inter..

[140] Zhang W., Chen J.L., Backman L.J., Malm A.D., Danielson P. (2017). Surface Topography and Mechanical Strain Promote Keratocyte Phenotype and Extracellular Matrix Formation in a Biomimetic 3D Corneal Model. Adv. Healthc. Mater..

[141] Song E., Chen K.M., Margolis M.S., Wungcharoen T., Koh W.G., Myung D. (2024). Electrospun Nanofiber Membrane for Cultured Corneal Endothelial Cell Transplantation. Bioengineering.

[142] Dong Y.H., Jaleh B., Ashrafi G., Kashfi M., Rhee K.Y. (2025). Mechanical properties of the hybrids of natural (alginate, collagen, chitin, cellulose, gelatin, chitosan, silk, and keratin) and synthetic electrospun nanofibers: A review. Int. J. Biol. Macromol..

[143] Kim J.I., Kim J.Y., Park C.H. (2018). Fabrication of transparent hemispherical 3D nanofibrous scaffolds with radially aligned patterns via a novel electrospinning method. Sci. Rep..

[144] Arabpour Z., Baradaran-Rafii A., Bakhshaiesh N.L., Ai J., Ebrahimi-Barough S., Esmaeili Malekabadi H., Nazeri N., Vaez A., Salehi M., Sefat F. (2019). Design and characterization of biodegradable multi layered electrospun nanofibers for corneal tissue engineering applications. J. Biomed. Mater. Res. Part A.

[145] Kong B., Sun W., Chen G.S., Tang S., Li M., Shao Z.W., Mi S.L. (2017). Tissue-engineered cornea constructed with compressed collagen and laser-perforated electrospun mat. Sci. Rep..

[146] Mahmood N., Sefat E., Roberts D., Gilger B.C., Gluck J.M. (2023). Application of Noggin-Coated Electrospun Scaffold in Corneal Wound Healing. Transl. Vis. Sci. Technol..

[147] Jafar H., Ahmed K., Rayyan R., Sotari S., Buqain R., Ali D., Al Bdour M., Awidi A. (2023). Plasma-Treated Electrospun PLGA Nanofiber Scaffold Supports Limbal Stem Cells. Polymers.

[148] Abdullah K.K., Molnár K. (2025). Current Trends and Future Prospects of Integrating Electrospinning With 3D Printing Techniques for Mimicking Bone Extracellular Matrix Scaffolds. J. Polym. Sci..

[149] Zhang Y.N., O’Mahony A., He Y., Barber T. (2024). Hydrodynamic shear stress’ impact on mammalian cell properties and its applications in 3D bioprinting. Biofabrication.

[150] Malekpour A., Chen X.B. (2022). Printability and Cell Viability in Extrusion-Based Bioprinting from Experimental, Computational, and Machine Learning Views. J. Funct. Biomater..

[151] Wang P.J., Sun Y.Z., Diao L.W., Liu H.T. (2024). Considering cell viability in 3D printing of structured inks: A comparative and equivalent analysis of fluid forces. Int. J. Bioprint..

[152] Wang Q.S., Liao Y.H., Ho Y.H., Wang K., Jin W.Z., Guan Y.M., Fu W.X. (2023). A study on cell viability based on thermal inkjet three-dimensional bioprinting. Phys. Fluids.

[153] Ouyang L.L., Yao R., Zhao Y., Sun W. (2016). Effect of bioink properties on printability and cell viability for 3D bioplotting of embryonic stem cells. Biofabrication.

[154] Boularaoui S., Shanti A., Khan K.A., Iacoponi S., Christoforou N., Stefanini C. (2022). Harnessing shear stress preconditioning to improve cell viability in 3D post-printed biostructures using extrusion bioprinting. Bioprinting.

[155] Basoz D., Akalinli A., Buyuksungur S., Celebi A.R.C., Yucel D., Hasirci N., Hasirci V. (2025). Preclinical Testing of 3D Printed, Cell Loaded Hydrogel Based Corneal Substitutes on Rabbit Model. Macromol. Biosci..

[156] He B.B., Wang J., Xie M.T., Xu M.Y., Zhang Y.H., Hao H.J., Xing X.L., Lu W., Han Q.H., Liu W.G. (2022). 3D printed biomimetic epithelium/stroma bilayer hydrogel implant for corneal regeneration. Bioact. Mater..

[157] Xu Y.N., Liu W.F., Zhao Q., Feng X.Y., Li Z.B., Huang Y.R., Liu J., Peng Y.H., Song W.J., Ren L. (2025). 3D bioprinted GelMA/collagen hydrogel for corneal stroma regeneration. Biofabrication.

[158] Ghosh A., Bera A.K., Ghosh S., Singh V., Basu S., Pati F. (2024). Bioprinting a resilient and transparent cornea stroma equivalent: Harnessing dual crosslinking strategy with decellularized cornea matrix and silk fibroin hybrid. Biofabrication.

[159] Pietryga K., Jesse K., Drzyzga R., Konka A., Zembala-John J., Kowalik A., Kielbowicz Z., Cwirko M., Buldak R.J., Dobrowolski D. (2024). Bio-printing method as a novel approach to obtain a fibrin scaffold settled by limbal epithelial cells for corneal regeneration. Sci. Rep..

[160] Isaacson A., Swioklo S., Connon C.J. (2018). 3D bioprinting of a corneal stroma equivalent. Exp. Eye Res..

[161] Bektas C.K., Hasirci V. (2020). Cell loaded 3D bioprinted GelMA hydrogels for corneal stroma engineering. Biomater. Sci..

[162] Moro A., Samanta S., Honkamaki L., Rangasami V.K., Puistola P., Kauppila M., Narkilahti S., Miettinen S., Oommen O., Skottman H. (2022). Hyaluronic acid based next generation bioink for 3D bioprinting of human stem cell derived corneal stromal model with innervation. Biofabrication.

[163] Gronroos P., Moro A., Puistola P., Hopia K., Huuskonen M., Viheriala T., Ilmarinen T., Skottman H. (2024). Bioprinting of human pluripotent stem cell derived corneal endothelial cells with hydrazone crosslinked hyaluronic acid bioink. Stem Cell Res. Ther..

[164] Li S., Zhang H., Sun L., Zhang X., Guo M., Liu J., Wang W., Zhao N. (2024). 4D printing of biological macromolecules employing handheld bioprinters for in situ wound healing applications. Int. J. Biol. Macromol..

[165] Kumar D., Malviya R., Sridhar S.B., Wadhwa T., Talath S., Shareef J. (2025). Trends in 4D Printed Shape Memory Biomaterials for Tissue Engineering Applications. Curr. Pharm. Des..

[166] Kalogeropoulou M., Díaz-Payno P.J., Mirzaali M.J., van Osch G.J.V.M., Fratila-Apachitei L.E., Zadpoor A.A. (2024). 4D printed shape-shifting biomaterials for tissue engineering and regenerative medicine applications. Biofabrication.

[167] Wang Z.B., Jiang C.Q., Fan Y.Q., Hao X.D., Dong Y.H., He X.J., Gao J.N., Zhang Y.C., Li M., Wang M.Y. (2024). The application of a 4D-printed chitosan-based stem cell carrier for the repair of corneal alkali burns. Stem Cell Res. Ther..

[168] Song W.L., Huang W.H., Qu J.Z., Xiao C.J., Yin H.N., Liu X.Z., Xu W.K. (2025). The application prospects of 4D printing tissue engineering materials in oral bone regeneration. Int. J. Bioprint..

[169] Stepanovska J., Otahal M., Hanzalek K., Supova M., Matejka R. (2021). pH Modification of High-Concentrated Collagen Bioinks as a Factor Affecting Cell Viability, Mechanical Properties, and Printability. Gels.

[170] Lameirinhas N.S., Teixeira M.C., Carvalho J.P.F., Valente B.F.A., Luís J.L., Duarte I.F., Pinto R.J.B., Oliveira H., Oliveira J.M., Silvestre A.J.D. (2025). Biofabrication of HepG2 Cells-Laden 3D Structures Using Nanocellulose-Reinforced Gelatin-Based Hydrogel Bioinks: Materials Characterization, Cell Viability Assessment, and Metabolomic Analysis. Acs Biomater. Sci. Eng..

[171] Limon S.M., Sarah R., Habib A. (2025). Integrating Decision Trees and Clustering for Efficient Optimization of Bioink Rheology and 3D Bioprinted Construct Microenvironments. J. Manuf. Sci. Eng..

[172] Zhang C.L., Elvitigala K.C.M.L., Mubarok W., Okano Y., Sakai S. (2024). Machine learning-based prediction and optimisation framework for as-extruded cell viability in extrusion-based 3D bioprinting. Virtual Phys. Prototyp..

[173] Shao L., Gao Q., Xie C.Q., Fu J.Z., Xiang M.X., He Y. (2020). Synchronous 3D Bioprinting of Large-Scale Cell-Laden Constructs with Nutrient Networks. Adv. Healthc. Mater..

[174] Wang Y.H., Xie C.N., Zhang Z.M., Liu H.N., Xu H.X., Peng Z.Y., Liu C., Li J.J., Wang C.Q., Xu T. (2022). 3D Printed Integrated Bionic Oxygenated Scaffold for Bone Regeneration. ACS Appl. Mater. Interfaces.

[175] Wang X.C., Yang C.Y., Yu Y.R., Zhao Y.J. (2022). In Situ 3D Bioprinting Living Photosynthetic Scaffolds for Autotrophic Wound Healing. Research.

[176] Guo B.J., Duan Y.C., Li Z.W., Tian Y., Cheng X.D., Liang C.X., Liu W.J., An B., Wei W.M., Gao T.T. (2023). High-Strength Cell Sheets and Vigorous Hydrogels from Mesenchymal Stem Cells Derived from Human Embryonic Stem Cells. ACS Appl. Mater. Interfaces.

